# Horizontally Acquired Quorum-Sensing Regulators Recruited by the PhoP Regulatory Network Expand the Host Adaptation Repertoire in the Phytopathogen *Pectobacterium brasiliense*

**DOI:** 10.1128/mSystems.00650-19

**Published:** 2020-01-28

**Authors:** Daniel Bellieny-Rabelo, Ntombikayise Precious Nkomo, Divine Yufetar Shyntum, Lucy Novungayo Moleleki

**Affiliations:** a Department of Biochemistry, Genetics and Microbiology, University of Pretoria, Pretoria, Gauteng, South Africa; b Forestry and Agricultural Biotechnology Institute, University of Pretoria, Pretoria, Gauteng, South Africa; University of California, Berkeley

**Keywords:** *Pectobacterium*, carbapenems, gene regulatory networks, horizontal gene transfer, quorum sensing, type III secretion systems, type VI secretion systems

## Abstract

In this study, we examine the impact of transcriptional network rearrangements driven by horizontal gene acquisition in PhoP and SlyA regulons using as a case study a phytopathosystem comprised of potato tubers and the soft-rot pathogen Pectobacterium brasiliense 1692 (Pb1692). Genome simulations and statistical analyses uncovered the tendency of PhoP and SlyA networks to mobilize lineage-specific traits predicted as horizontal gene transfer at late infection, highlighting the prominence of regulatory network rearrangements in this stage of infection. The evidence further supports the circumscription of two horizontally acquired quorum-sensing regulators (*carR* and *expR1*) by the PhoP network. By recruiting *carR* and *expR1*, the PhoP network also impacts certain host adaptation- and bacterial competition-related systems, seemingly in a quorum sensing-dependent manner, such as the type VI secretion system, carbapenem biosynthesis, and plant cell wall-degrading enzymes (PCWDE) like cellulases and pectate lyases. Conversely, polygalacturonases and the type III secretion system (T3SS) exhibit a transcriptional pattern that suggests quorum-sensing-independent regulation by the PhoP network. This includes an uncharacterized novel phage-related gene family within the T3SS gene cluster that has been recently acquired by two *Pectobacterium* species. The evidence further suggests a PhoP-dependent regulation of carbapenem- and PCWDE-encoding genes based on the synthesized products’ optimum pH. The PhoP network also controls *slyA* expression *in planta*, which seems to impact carbohydrate metabolism regulation, especially at early infection, when 76.2% of the SlyA-regulated genes from that category also require PhoP to achieve normal expression levels.

**IMPORTANCE** Exchanging genetic material through horizontal transfer is a critical mechanism that drives bacteria to efficiently adapt to host defenses. In this report, we demonstrate that a specific plant-pathogenic species (from the *Pectobacterium* genus) successfully integrated a population density-based behavior system (quorum sensing) acquired through horizontal transfer into a resident stress-response gene regulatory network controlled by the PhoP protein. Evidence found here underscores that subsets of bacterial weaponry critical for colonization, typically known to respond to quorum sensing, are also controlled by PhoP. Some of these traits include different types of enzymes that can efficiently break down plant cell walls depending on the environmental acidity level. Thus, we hypothesize that PhoP’s ability to elicit regulatory responses based on acidity and nutrient availability fluctuations has strongly impacted the fixation of its regulatory connection with quorum sensing. In addition, another global gene regulator, known as SlyA, was found under the PhoP regulatory network. The SlyA regulator controls a series of carbohydrate metabolism-related traits, which also seem to be regulated by PhoP. By centralizing quorum sensing and *slyA* under PhoP scrutiny, *Pectobacterium* cells added an advantageous layer of control over those two networks that potentially enhances colonization efficiency.

## Abstract (precis)

In this study, we examine the impact of transcriptional network rearrangements driven by horizontal gene acquisition in PhoP and SlyA regulons using as a case study a phytopathosystem comprised of potato tubers and the soft-rot pathogen Pectobacterium brasiliense 1692 (Pb1692).

## INTRODUCTION

Highly specialized colonization traits are constantly selected in phytopathogenic Gram-negative bacteria to overcome severe obstacles imposed by plant apoplasts. The apoplastic space comprises a nutrient-poor milieu that harbors an extensive inventory of toxic and defense-related molecules, such as plant antimicrobial peptides, reactive oxygen species, and plant organic compounds (1, 2). In addition, the apoplastic space is generally acidic (ranging between pH 4.5 and 6.5) due to the presence of organic acids such as malic and citric acids, which also enforces the anti-growth strategy against pathogenic invasions (3). In this context, a crucial regulatory system in bacteria, which is frequently associated with response to acidic stress, is the PhoQ/PhoP two-component system (4). This response-based system is highly conserved and widely studied across bacterial lineages because of its prominent role as a global transcriptional regulator.

The simultaneous flow of transcription/translation in bacterial cells inhabiting such competitive environments requires highly optimized regulatory mechanisms to ensure accurate responses during colonization. Thus, to cope with frequent gene gains and losses that result mainly from horizontal transfer events, bacterial regulatory circuits must efficiently accommodate newly acquired genes (5). Horizontal gene transfer is perceived as a critical driving force in the evolution of bacterial genomes from which a number of pathogenicity themes emerge, including some secretion systems and prophages (6, 7). Hence, it is not surprising that the influence of important virulence regulators such as SlyA (8) or PecS (9) over regions incorporated through horizontal gene transfer (HGT) have been previously reported. Both SlyA and PecS belong to the MarR family of transcriptional regulators, which encompasses a range of genes involved in virulence and antibiotic resistance control (10–12).

Among Gram-negative bacteria, one specific group, commonly referred to as soft-rot *Pectobacteriaceae* (SRP) (13, 14) (formerly known as soft-rot *Enterobacteriaceae*), has increasingly gained attention over the last few decades as causative agents of wilt/blackleg diseases in economically important crops worldwide (15, 16). This group is most prominently represented by *Pectobacterium* and *Dickeya* genera. The SRPs are opportunistic Gram-negative pathogens capable of producing distinctly larger amounts of pectinolytic enzymes than other pectolytic bacteria (e.g., *Clostridium* spp., *Bacillus* spp., and *Pseudomonas* spp.) (17). These plant cell wall-degrading enzymes (PCWDEs) encompass a variety of families that concertedly promote disease through tissue maceration (18). While some PCWDE classes exhibit close to neutral or high optimum pH, such as cellulases and pectate and pectin lyases (Cel, Pel, and Pnl), others function at low optimum pH, namely, polygalacturonases (Peh) (19, 20). In this sense, the expression of different groups of PCWDEs tends to be regulated according to the pH within the plant tissue, which is acidic in the apoplast at first and then becomes basic as the disease progresses (17). In this context, one of the best-characterized mechanisms of PCWDE regulation in SRP pathogens is quorum sensing (QS) (21). This is a crucial regulatory circuit controlling population density-based behavior in SRP as well as in a large spectrum of bacteria (22, 23). Quorum-sensing networks in Gram-negative bacteria rely on recognition of autoinducer molecules, such as acyl-homoserine lactones (AHL) and other products synthesized using *S*-adenosylmethionine, by specialized receptors such as ExpR/LuxR homologs (24, 25). The complexed form, ExpR/LuxR-autoinducer, then can bind specific DNA regions to participate in transcriptional regulation (26). One of the regions regulated by the ExpR-autoinducer complex is the *rsmA* promoter region. Thereafter, the transcriptional repression of *rsmA* prevents RsmA-induced inactivation of PCWDEs (27). In addition to PCWDEs, QS has been shown to regulate other traits involved in virulence and interbacterial competition, such as the carbapenem antibiotic (*car* genes) and the type III and VI secretion systems (T3SS and T6SS) in *Pectobacterium* spp. (21, 28).

In the present report, we investigate the impact of transcriptional network rearrangements in two global transcriptional regulators (PhoP and SlyA) on the *in planta* regulation of crucial host adaptation- and fitness-oriented systems. Since regulatory network rearrangement events have the potential to give rise to phenotypic differences in pathogenesis development among these organisms, understanding these processes is crucial to unveiling how these networks shape pathogenicity in different lineages.

## RESULTS

### RNA-Seq mapping and the reach of PhoP and SlyA regulons *in planta*.

To examine both PhoP and SlyA regulatory networks *in planta*, we engineered Pb1692Δ*slyA* and Pb1692Δ*phoP* mutants using the lambda recombination technique (see Materials and Methods for details). The integrity of the mutants and complement strains were confirmed by PCR analyses (see Fig. S1A and B in the supplemental material) as well as by DNA sequencing. Additionally, an *in vitro* growth assay demonstrated that the deletion of neither the *phoP* nor *slyA* gene impaired the growth of the mutant strains (Fig. S1C and D). The global impact caused by *phoP* or *slyA* deletion in the Pb1692 genome toward *in planta* transcriptional profiles next was analyzed in original whole-transcriptome data sets comprising ∼124 million transcriptome sequencing (RNA-Seq) reads (Table S1). By comparing significant gene expression changes between the wild type and either the Pb1692Δ*slyA* or Pb1692Δ*phoP* mutant, we infer transcriptional regulation affected by these regulatory networks (see Materials and Methods for details). Additional validation by quantitative reverse transcription-PCR (qRT-PCR) of the differentially expressed genes of particular interest for this study was also conducted (Fig. S2). The samples selected for this study depicted two stages of plant infection, 12 and 24 h postinfection (hpi), here denoted early (EI) and late (LI) infection, respectively. The data set exhibited good quality, as 89.5% (∼111 million) of the reads were mapped on the Pb1692 reference genome (Table S1). Of these, 89% (∼99 million reads) were uniquely mapped on the reference genome. Transcriptional variations between wild-type Pb1692 and mutant strains during infection enabled collective identification of 480 genes affected (up-/downregulation; see Materials and Methods for details) either by PhoP or SlyA regulatory networks at the two time points (Table S2). In this context, the prominent transcriptional reach of PhoP compared to that of SlyA during plant infection was observed, as the former directly or indirectly controls 410 genes but the latter controls 145.

10.1128/mSystems.00650-19.1 FIG S1 PCR amplicons used to generate the Pb1692Δ*phoP* and Pb1692Δ*slyA* mutants and growth curves of Pb1692 mutant strains. (A) Lane 1, DNA ladder; 2, *phoP* downstream PCR fragment; 3, kanamycin cassette PCR product; 4, *phoP* upstream PCR fragment; 5, fusion product consisting of the downstream fragment, kanamycin cassette, and upstream fragments; 5, fusion product consisting of the downstream fragment, kanamycin cassette, and upstream fragments; 6 and 7, Km1 and Km2 primers are internal kanamycin primers. (B) Lane 1, DNA ladder; 2, *slyA* downstream PCR fragment; 3, kanamycin cassette PCR product; 4, *slyA* upstream PCR fragment; 5, fusion product consisting of the downstream fragment, kanamycin cassette, and upstream fragments; 6, Km1 primers and internal kanamycin primers. (C) Growth and survival of Pb1692 wild-type, Pb1692Δ*phoP*, and Pb1692Δ*phoP*-p*phoP* strains in LB broth at 37°C for 16 h, with agitation at 370 rpm. Data points represent means from three biological replicates. (D) Growth and survival of Pb1692 wild-type, Pb1692Δ*slyA*, and Pb1692Δ*slyA*-p*slyA* strains in LB broth at 37°C for 16 h, with agitation at 370 rpm. Data points represent means from three biological replicates. Download FIG S1, TIF file, 1.2 MB. Copyright © 2020 Bellieny-Rabelo et al. 2020 Bellieny-Rabelo et al. This content is distributed under the terms of the Creative Commons Attribution 4.0 International license.

10.1128/mSystems.00650-19.2 FIG S2 Additional validation through qRT-PCR of differentially expressed genes obtained from RNA-Seq. The relative transcript abundance was compared between mutant (Δ*phoP* or Δ*slyA*) and wild-type (wt) strains of Pb1692 through qRT-PCR. Statistical significance between wt and mutant strains for each gene was obtained through one-tailed *t* test: *, *P* > 0.05; **, *P* > 0.01. Genes annotated in the carbohydrate metabolism category (KEGG 09101) are represented as *AED-0001174*, *celC* (*AED-0001290*), and *bgl*F (*AED-0003729*). Genes associated with plant cell wall degradation (*peh*A, *AED-0002061*; *peh*N, *AED-0000675*), T6SS (*tss*E, *AED-0001996*; *tss*C, *AED-0001994*), T3SS (*bigA*, *AED-0003294*; *hrpV*, *AED-0001197*), and transcriptional regulation (*expR1*, *AED-0000069*; *carR*, *AED-0003542*; *deo*R, *AED-0001096*) are represented by the gene names annotated through homology prediction using the eggNOG database. Download FIG S2, TIF file, 0.4 MB. Copyright © 2020 Bellieny-Rabelo et al. 2020 Bellieny-Rabelo et al. This content is distributed under the terms of the Creative Commons Attribution 4.0 International license.

10.1128/mSystems.00650-19.4 TABLE S1 Mapping summary of RNA-Seq reads from mutant strains on Pb1692 reference genome. Download Table S1, XLSX file, 0.01 MB. Copyright © 2020 Bellieny-Rabelo et al. 2020 Bellieny-Rabelo et al. This content is distributed under the terms of the Creative Commons Attribution 4.0 International license.

10.1128/mSystems.00650-19.5 TABLE S2 PhoP and SlyA *in planta* regulons obtained from whole-transcriptome data sets obtained during early (12 h postinfection [hpi]) or late (24 hpi) infection of Pb1692. Download Table S2, XLSX file, 0.1 MB. Copyright © 2020 Bellieny-Rabelo et al. 2020 Bellieny-Rabelo et al. This content is distributed under the terms of the Creative Commons Attribution 4.0 International license.

**FIG 1:**
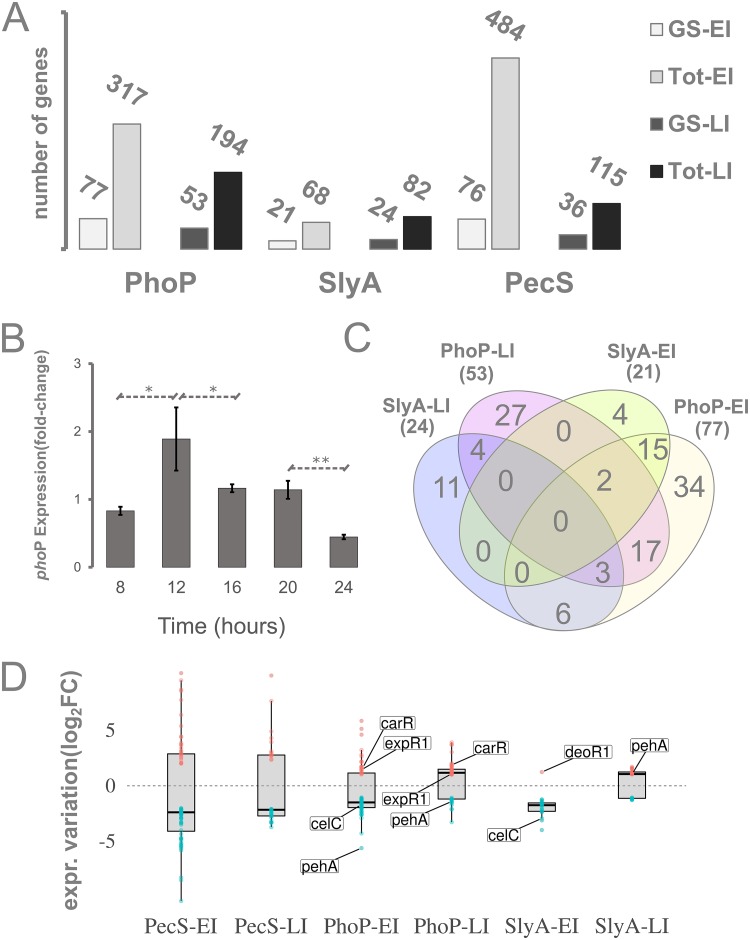
Genome-wide regulatory impact of three global regulators in Pb1692 and Dd3937 and the transcriptional levels of *phoP* regulator during plant infection. (A) Genus-specific (GS) versus total gene (Tot) amounts in PhoP, SlyA, and PecS regulons *in planta* both at early and late infection (EI and LI) are represented in the bar plot. PhoP and SlyA regulons were extracted from Pb1692 infection samples. PecS regulons were extracted from Dd3937 samples. (B) Transcriptional levels of the *phoP* gene measured by qRT-PCR in 4-h intervals in potato tubers infected by Pb1692 across the first 24 hpi. Consecutive time points were tested for statistically significant (*t* test) differences in their expression levels. Significant results are marked with asterisks in the graph (*, *P < *0.05; **, *P < *0.005). (C) Venn diagrams depicting genus-specific genes found in PhoP and SlyA *in planta* regulons at EI and LI assessed in Pb1692. The total number of genus-specific genes found within each regulon is highlighted under each regulon label. (D) Gene transcriptional variations (log_2_ fold change) are depicted specifically for genus-specific genes. Transcriptional variation data were extracted from PhoP, SlyA, and PecS regulons either at early or late infection (EI and LI). The box plots are overlaid with dot plots, in which each dot represents a single gene expression value. In PhoP and SlyA regulons, specific genes are labeled for polygalacturonase A (*pehA*, *AED-0002061*), quorum-sensing regulators (*carR*, *AED-0003542*; *expR1*, *AED-0000069*), a transcriptional regulator (*deoR1*, *AED-0001096*), and a PTS permease gene (*celC*, *AED-0001290*).

From the PhoP regulon, we detected a 36.1% reduction in the total network size between early and late infection (Fig. 1A). This phenomenon could result from a variety of non-mutually exclusive events, among which the most predictable would be (i) increased number of active transcriptional regulators at 24 hpi competing with PhoP for promoter binding regions, (ii) PhoP dephosphorylation by PhoQ following the absence of triggering conditions, or (iii) decreased transcription of the *phoP* gene. Thus, to further inspect this shift in PhoP network sizes between EI and LI, we set out to evaluate the transcriptional variation of the *phoP* gene *in planta* by the Pb1692 wild type. First, Pb1692 was inoculated into potato tubers and samples were harvested at 8, 12, 16, 20, and 24 hpi. Next, qRT-PCR was used to analyze the expression of the *phoP* gene in those samples. The results showed that the relative expression of the *phoP* gene nearly doubled between 8 and 12 hpi (Fig. 1B). This increase was followed by a significant decrease (*P* = 0.042) between 12 and 16 hpi. After keeping a constant expression level between 16 and 20 hpi, Pb1692 cells drastically decrease *phoP* gene expression at 24 hpi (*P* = 0.004). This converges with the shrinkage observed in the network size. Hence, since PhoP is frequently associated with transcriptional responses to acidic environments and low Mg^2+^ concentration, the elevated *phoP* transcription in the early stages of infection is arguably a direct response to the nutrient-poor environment found in the apoplast. Conversely, after a certain time point between 20 and 24 hpi, *phoP* transcription decreases, which is consistent with the reduced cellular demand for this regulator following environmental alkalization due to progressive host cell lysis.

### Determining the genus-specific content in PhoP, SlyA, and PecS *in planta* regulons.

The prevalence of distinctive traits (genus specific) next was inspected in the full range of transcriptionally regulated genes by three global regulators frequently associated with virulence and/or stress responses. This was achieved by adding the PecS regulon data set to this analysis, which was the only publicly available *in planta* whole-transcriptome data set obtained from an SRP mutant strain lacking a transcriptional regulator (9). The PecS regulon was obtained from Dickeya dadantii strain 3937 (Dd3937). We then analyzed 6 distinct regulons obtained from those 3 global regulators in two different stages (i.e., early and late) of plant infection. Hence, this analysis focuses on three early infection regulons, i.e., PhoP-, SlyA-, and PecS-EI, along with three late infection regulons, i.e., PhoP-, SlyA-, and PecS-LI. The identification of distinctive systems between the two genera was achieved by predicting genus-specific genes in *Pectobacterium* and *Dickeya* genera based on protein sequence orthology. This analysis was underpinned by an extensive correlational database previously generated that included 39 and 61 strains from *Dickeya* and *Pectobacterium* genera, respectively (6). By using this database, we inquired, for each gene in a given strain, how many orthologs could be detected in other strains within the genus as well as in the opposing genus. Those gene products for which no orthologous counterparts exist in the opposing genus were assigned, given the scope of this study, as GS in the SRP context, i.e., *Pectobacterium* exclusive or *Dickeya* exclusive.

The presence of GS protein-coding genes next was surveyed within each of the *in planta* regulons. The preliminary aim was to measure the relative presence of GS genes in these regulons in specific infection stages. Our screening revealed that across the three early infection regulons (from PhoP, SlyA, and PecS), 15 to 31% of the regulated genes are GS. Interestingly, at late infection, two out of three regulons (except for SlyA) exhibited increased relative GS content, ranging between 27 and 31% (Fig. 1A). Notably, the PecS regulons exhibited the most drastic shift in the GS content proportion between EI (15%) and LI (31%). Furthermore, by comparing the GS gene sets of SlyA and PhoP regulons from Pb1692, the presence of GS genes that are exclusive to individual regulons (regulon exclusive) was more conspicuous in those of late infection. Thus, in PhoP and SlyA data sets, 46 to 51% (27/53 and 11/24) of the GS genes regulated at LI are regulon exclusive (Fig. 1C). Conversely, 19 to 44% of the GS (4/21 and 34/77) were regulon exclusive among EI samples. This analysis revealed that at late infection, PhoP, SlyA (in Pb1692), and PecS (in Dd3937) tend to mobilize transcription of an equal or greater proportion of GS genes than during early infection. Additionally, it also showed that PhoP and SlyA exhibit larger relative amounts of GS regulon-exclusive genes at late infection than during early infection.

In the SlyA-EI data set, the only GS gene displaying increased expression was a MarR transcriptional regulator homolog to *deoR1* (*AED-0001096*), which is frequently associated with repression of carbohydrate metabolism (Fig. 1D). Interestingly, the *deoR1* homolog in Pb1692 is regulon exclusive from SlyA-EI. Furthermore, besides promoting activation of *deoR1*, the absence of SlyA also causes significant repression over several sugar permeases, including the GS gene *celC* (*AED-0001290*). All of these permeases also seem to be affected by *phoP* deletion in early stages of infection. This indicates a particular demand for the transcriptional regulation of certain carbohydrate metabolism-related systems controlled by *slyA* and *deoR1* regulators in specific stages of infection.

Moreover, a *Pectobacterium*-exclusive gene encoding a polygalacturonase (Peh) was found within PhoP *in planta* regulons (Fig. 1D). Converging with the evidence for the transcriptional regulation of this PCWDE-encoding gene, especially by PhoP, we also found two QS regulators under PhoP control, namely, *expR1* and *carR* (Fig. 1D). These QS regulators are GS genes in *Pectobacterium* genomes. While ExpR1 is often, but not exclusively, associated with transcriptional regulation of pectinolytic enzymes (21, 29), CarR is associated with regulation of carbapenem antibiotic biosynthesis (28). The presence of *luxR-expR* homologs within the PhoP network might be directly linked to the presence of two bacterial secretion systems. This type of association may directly impact fitness, which will be inspected in a subsequent section.

### Horizontal acquisitions in genus-specific contents and their regulation by PhoP, SlyA, and PecS.

While the presence of GS genes across the six regulons was observed, their origin was still unclear. The GS genes observed here may have emerged through different processes, such as (i) gene loss in the neighboring lineage, (ii) intralineage functional divergence following gene duplication, or (iii) gene acquisition via HGT. Hence, aiming to evaluate the prevalence of transcriptional network rearrangement processes, especially in Pb1692 networks, we set out to predict HGT candidates using parametric methods. Per previous observations (30), we chose a gene-based method (GC content at third codon position [GC3]) and a window-based method (dinucleotide frequencies [DINT_KL]) to evaluate sequence composition bias in Pb1692 and Dd3937 genes (see Materials and Methods for details). These predictions will be further compared to the orthology-based prediction of genus-specific genes as an additional layer of evidence.

By examining the entire set of GS genes in Pb1692 (787 genes), we found that 36.2 and 19.3%, respectively, were predicted as HGT candidates by GC3 or DINT_KL methods. Importantly, 53% of the high-confidence HGT candidates, meaning those supported by both GC3 and DINT_KL, belong to the GS gene set. On the other hand, in Dd3937 37.8% of the high-confidence HGT candidates are GS genes. This preliminary inquiry revealed that the GS content in Pb1692 tends to include a larger portion of horizontally acquired genes than Dd3937. Furthermore, the inspection of PecS *in planta* regulons at early and late infection in Dd3937 revealed that 6.6% and 44.4%, respectively, of the GS genes are high-confidence HGT candidates. On the other hand, by examining PhoP and SlyA *in planta* regulons in Pb1692, the high-confidence candidates within the GS genes accounted for 24.7% and 14.3%, respectively, at early infection and 30.2% and 37.5%, respectively, at late infection. In this context, the genus-specific HGT candidate (GS-HGT) genes most likely arise from recent gene acquisitions in a given genus. Hence, this preliminary analysis supports that the regulation of some distinctive traits observed among *Pectobacterium* and *Dickeya* genera involves recent rearrangements in PhoP, SlyA, and PecS regulatory networks driven by HGT.

**FIG 2:**
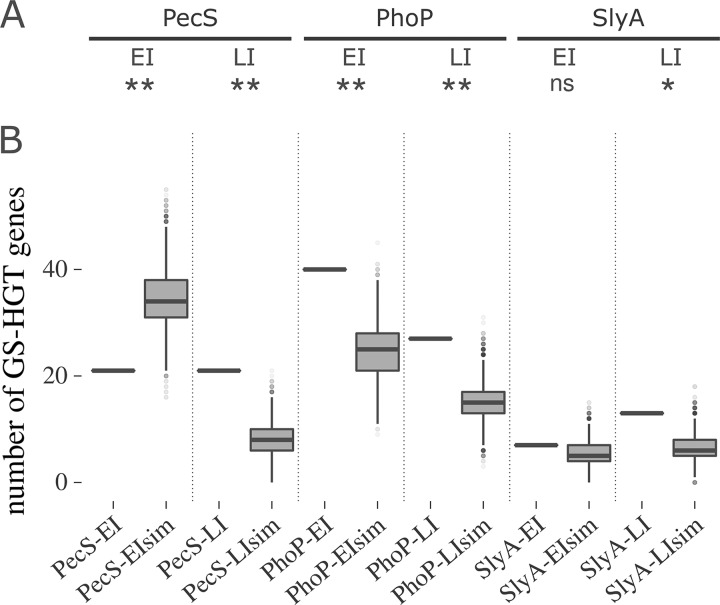
Overrepresentation of genus-specific HGT candidate genes in PhoP, SlyA, and PecS regulons in Pb1692 and Dd3937. (A) The significance of genus-specific HGT candidate (GS-HGT) gene occurrence in each regulon was analyzed through two-tailed Fisher exact tests using the respective species genomes as the background set (*, *P < *0.05; **, *P < *0.01; nonsignificant [ns], *P > *0.05). (B) Each section of the plot (separated by dotted lines) represents the comparison between the actual number of GS-HGT genes found in each regulon as a single horizontal line and the distribution of GS-HGT gene amounts found in each of the 10,000 computationally shuffled versions of the respective regulons (see Materials and Methods for details). The PecS data sets were obtained from Dd3937, whereas PhoP and SlyA data sets were obtained from Pb1692. The simulated data sets are labeled with the Sim postfix in the graph.

To inspect whether these regulatory networks are biased toward or against controlling GS-HGT candidate genes, we conducted a statistical enrichment analysis. The results from Fisher’s exact tests showed that, for the three LI regulons analyzed (from PhoP, SlyA, and PecS), there is an overrepresentation of GS-HGT genes (Fig. 2A). Among them, PhoP is the only regulator exhibiting overrepresentation of GS-HGT genes both on EI and LI regulatory networks. Curiously, the PecS-EI regulon exhibits a negative correlation with GS-HGT genes. We next aimed to further confirm these results using a different approach. Computational simulations were performed to shuffle the genomes from each organism (i.e., Pb1692 and Dd3937), generating pseudogenomes. This strategy aims to empirically evaluate the frequency at which GS-HGT genes should be expected by chance in these regulons and compare this with the real data. For each of the six regulons analyzed, this experiment generated 10,000 shuffled pseudogenomes (see Materials and Methods for details). The results corroborated, by empirical means, what statistical analysis had previously established (Fig. 2B). Overall, these analyses showed that transient shifts, in this case due to temporal variation, in regulon profiles can alter their bias toward the presence of recently acquired genes. Although at late infection all regulons exhibited a consistent bias toward the regulation of GS-HGT genes, only one of them (PhoP) displayed similar bias at early infection. On the contrary, the PecS network seemed to avoid transcriptional interactions with GS-HGT genes at early infection. Overall, this evidence suggests a particular requirement for expression of distinctive traits seemingly acquired through HGT in these organisms at late stages of infection.

### Host adaptation and regulatory systems acquired by PhoP and SlyA networks.

**FIG 3:**
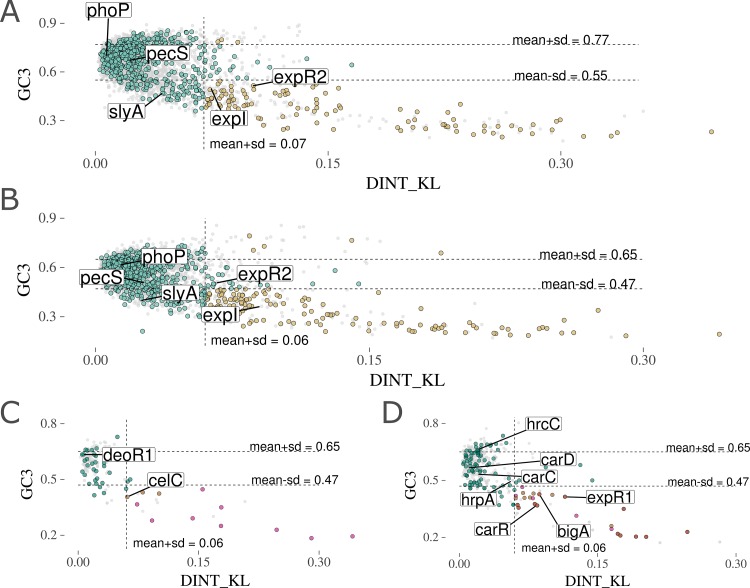
Horizontal gene transfer predictions for genus-specific genes in Dd3937 and Pb1692. (A) Two sequence composition measurements methods are represented for each gene in the Dd3937 genome as GC content at the third codon position index (GC3) in the *y* axis and the Kullback-Leibler distance of dinucleotide frequency distributions (DINT_KL) relative to the Dd3937 genome in the *x* axis. The coding sequences from Dd3937 comprise the background set, colored gray. From this set, specific genes are colored according to both the orthologous conservation in SRP genomes and sequence composition distance to the Dd3937 genome in both GC3 and DINT_KL metrics. Those genes exhibiting above-threshold (means ± SD) values are represented in light brown if they are genus specific (orthologs found in *Dickeya* but not in *Pectobacterium* genomes). Those genes exhibiting below-threshold values in either GC3 or DINT_KL are represented in green. Five genes of interest in this study are the following: *pec*S (*DDA3937_RS20885*), *phoP* (*DDA3837_RS11500*), *slyA* (*DDA3937_RS12595*), *expR2* (*DDA3937_RS20730*), and *expI* (*DDA3937_RS20725*). (B) The graph follows the same representation as that described for panel A. All analyses presented above for Dd3937 and the *Dickeya* genus are mirrored for Pb1692 and the *Pectobacterium* genus in panel B. Different functional classes are labeled as transcriptional regulators *pec*S (*AED-0003935*), *phoP* (*AED-0004376*), *slyA* (*AED-0001000*), *expR2* (*AED-0002741*), and *expI* (*AED-0000062*). (C) The entire set of genes regulated *in planta* (at early or late infection) by SlyA in Pb1692 comprises the background set, colored gray. Genus-specific genes are highlighted in green if below threshold, light brown if above threshold and regulated at early infection, pink if above threshold and regulated at late infection, or dark brown if above threshold and regulated at both early and late infection. Genes associated with carbohydrate metabolism regulation are *celC* (*AED-0001290*) and *deoR1* (*AED-0001096*). (D) The graph follows the representation described for panel C but depicting PhoP regulons (early and late infection) in Pb1692. Genes from different Pb1692 operons are the following: type III secretion operon, *hrpA* (*AED-0001214*), *hrcC* (*AED-0003292*), and *bigA* (*AED-0003294*); carbapenem biosynthesis associated, *carR* (*AED-0003542*), *carD* (*AED-0003538*), and *carC* (*AED-0003539*); and the transcriptional regulator *expR1* (*AED-0000069*).

Highly conserved genes among the SRPs, such as *phoP* (*AED-0004376* in Pb1692, *DDA3937_RS11500* in Dd3937) or *pec*S (*AED-0003935* and *DDA3937_RS20885*), exhibited overall sequence composition similar to that of their resident genomes (Dd3937 or Pb1692) (Fig. 3A and B and Table S3). Unlike these two regulators, *slyA* (*AED-0001000* and *DDA3937_RS12595*) homologs consistently exhibited deviant GC3 indexes compared to those of the two resident genomes analyzed, although in terms of dinucleotide frequencies a significant distance was not observed. Thus, considering the conservation of *slyA* across all SRP genomes, it is likely that the acquisition of this gene occurred at least in the last common ancestor between *Pectobacterium* and *Dickeya* genera. We next observed that the SlyA-regulated GS transcriptional regulator *deoR1* (*AED-0001096*) had no support from HGT prediction (Fig. 3C). This indicates that this gene was lost in the *Dickeya* lineage, whereas most of the *Pectobacterium* retained it. In contrast, the SlyA-regulated gene involved in carbohydrate uptake, *celC* (*AED-0001290*), was predicted as an HGT candidate with high confidence. This indicates that the *Pectobacterium* genus benefits from increasing the regulatory complexity of carbohydrate metabolism either through gene gain or by simply keeping ancestral genes in the lineage. This notion is further corroborated by the enrichment of genes associated with carbohydrate metabolism in the SlyA-EI regulon (Fig. S3).

10.1128/mSystems.00650-19.3 FIG S3 Distribution of KEGG terms in the SlyA regulon at early infection. The best represented KEGG terms within the SlyA regulon at early infection are depicted in the graph according to their relative proportion (percent) of the entire genome on a log_2_ normalized scale. A gap in the graph separates the categories found as enriched according to the adjusted *P* value from Fisher exact tests at the bottom: *, FDR of <0.05. Download FIG S3, TIF file, 0.4 MB. Copyright © 2020 Bellieny-Rabelo et al. 2020 Bellieny-Rabelo et al. This content is distributed under the terms of the Creative Commons Attribution 4.0 International license.

10.1128/mSystems.00650-19.6 TABLE S3 HGT prediction of genes of interest in Pb1692 and Dd3937 based on two parametric methods (dinucleotide frequencies and GC3 content). Download Table S3, XLSX file, 0.01 MB. Copyright © 2020 Bellieny-Rabelo et al. 2020 Bellieny-Rabelo et al. This content is distributed under the terms of the Creative Commons Attribution 4.0 International license.

As for quorum-sensing regulators, the *expR2-virR* homologs showed stronger support for horizontal acquisition in Dd3937 (*DDA3937_RS20730*), supported by both GC3 and DINT_KL, than in Pb1692 (*AED-0002741*), which indicates that *Pectobacterium* and *Dickeya* lineages acquired *expR2* independently (Fig. 3A and B). This hypothesis will be further discussed in a subsequent section. Moreover, the *Pectobacterium*-exclusive PhoP-regulated transcriptional regulator *expR1* (*AED-0000069*) was strongly supported as a horizontal acquisition by both parametric methods in Pb1692 (Fig. 3D). This result is further corroborated by a similar profile observed in the Pb1692 *expI* gene (*AED-0000062*) (Fig. 3A and B). Furthermore, the carbapenem biosynthesis transcriptional regulator *carR* (*AED-0003542*) is another GS gene in Pb1692 that has been successfully supported as an HGT candidate with high confidence (Fig. 3D). In addition, *carR* not only apparently has been horizontally transferred into the Pb1692 genome but also is the only gene in the carbapenem biosynthesis gene cluster supported by both parametric methods for HGT prediction (Table S3). *carR* is also the only GS gene in the *car* gene cluster (Table S2). These results strongly suggest that *carR* was acquired by Pb1692 independently (probably at a later stage) from *carABCDEF* genes. The presence of two QS regulators (*expR1* and *carR*), along with two conspicuous genomic regions encoding bacterial secretion systems (i.e., T3- and T6SS) under PhoP regulation in Pb1692, also may be correlated. Hence, we conducted an in-depth investigation of the PhoP-dependent regulation of these secretion systems and the phenotypic outcomes that this regulatory association may give rise to.

### PhoP-dependent regulation of secretion systems and their effects on virulence and fitness.

**FIG 4:**
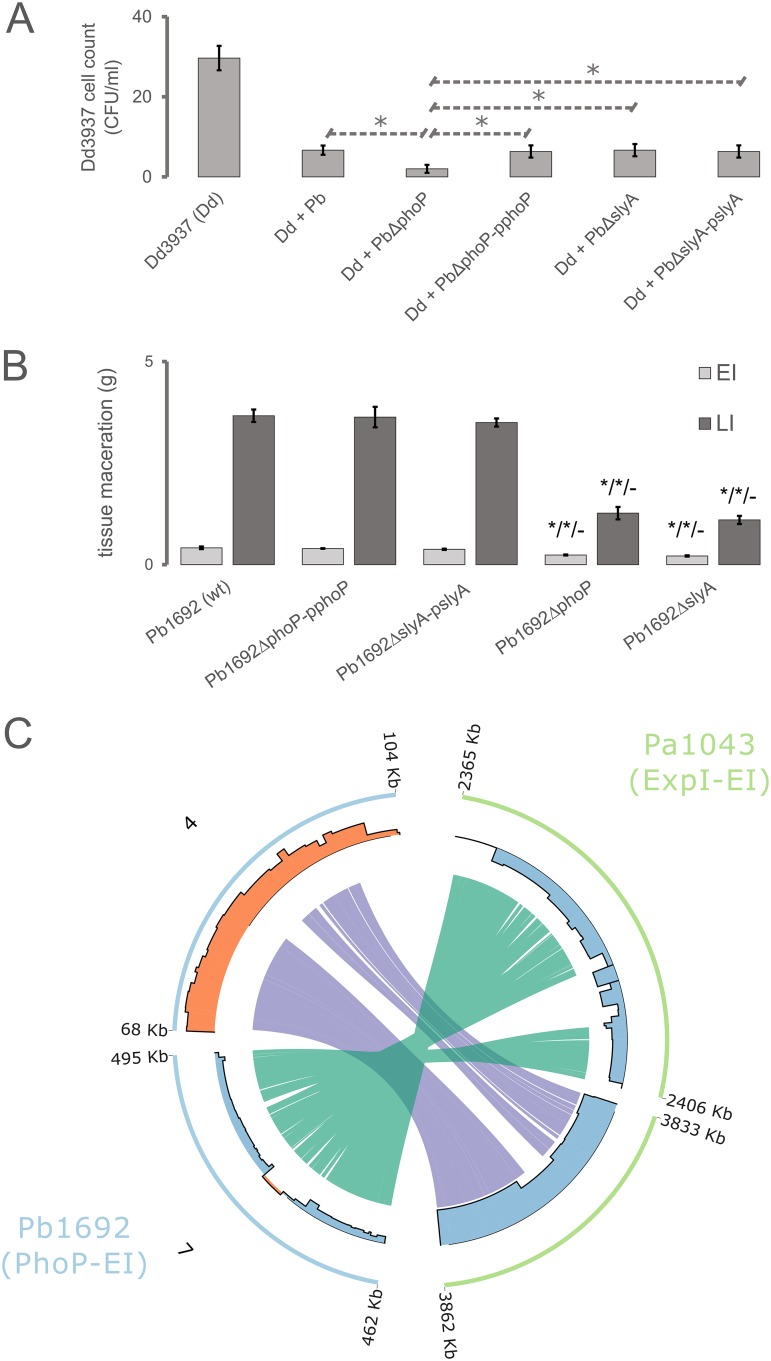
*In planta* bacterial competition and virulence of Pb1692 strains and the transcriptional variation of type III and VI secretion systems in *phoP* and *expI* mutants in Pb1692 and *Pa*1043, respectively. (A) The graph depicts the survival of Dickeya dadantii strain 3937 (Dd3937 or Dd) in potato tubers when coinoculated with different strains of *Pectobacterium brasiliense* strain 1692 (*Pb*), *Pb*Δ*phoP*, *Pb*Δ*slyA* (mutants), *P. brasiliense* (wild type), *Pb*Δ*phoP*-p*phoP*, and *Pb*Δ*slyA*-p*slyA* (complemented mutant strains), measured in CFU per milliliter. Significant differences between different samples were analyzed using Student's *t* test (*, *P < *0.05). (B) The weighed macerated tissue from potato tubers infected with Pb1692 strains was measured and the mass is expressed in grams. The strains (labels) analyzed are Pb1692 wild type (wt), Pb1692 *phoP* mutant (Pb1692Δ*phoP*), Pb1692 *slyA* mutant (Pb1692Δ*slyA*), Pb1692 *phoP* mutant complemented with *phoP* (Pb1692Δp*phoP*), and Pb1692 *slyA* mutant complemented with *slyA* (Pb1692Δp*slyA*). Above the mutant’s measurement bars, the sequence of three Student's *t* test results is depicted in which the mutants’ macerated masses are compared with that of the wild type, respective complement, and other mutant (the Pb1692Δ*phoP* mutant is compared to the Pb1692Δ*slyA* mutant and vice versa). (C) Genomic regions from Pb1692 (light blue) and *P. atrosepticum* strain scri1043 (*Pa*1043) (light-green) are represented in circular ideograms. Coordinates of 5′ and 3′ ends of each genomic region are displayed in kilobases. In the Pb1692 ideograms, respective contig numbers are labeled outside the ideogram. In the inner radius, the bar plot indicates, for each region, the transcriptional variation level (log_2_ fold change) found in each transcriptome study (either Liu et al. [21] for *Pa*1043 or this study for Pb1692). The bars are colored to highlight upregulation (orange) or downregulation (blue). The inner links binding genomic regions represent the homologous range of type VI (purple) and III (green) secretion systems conserved between Pb1692 and *Pa*1043 genomes.

The PhoP-dependent regulation of *Pectobacterium*-exclusive QS regulators, along with type III and VI secretion systems, prompted us to measure the ability of Pb1692Δ*phoP* to (i) outcompete other SRP *in planta* and (ii) cause tissue maceration in potato tubers. The Pb1692Δ*slyA* strain was also included for comparative means. Therefore, we first performed *in planta* bacterial competition assays to determine the relative contribution of PhoP to bacterial competition. For this experiment, Dd3937 was selected because it is a well-studied niche competitor of Pb1692 in potato tubers. The growth of Dd3937 when coinoculated *in planta* with the Pb1692Δ*phoP* mutant displayed a 5-fold reduction (in CFU per milliliter), as opposed to a 3-fold reduction when coinoculated with wild-type Pb1692 (Fig. 4A). Conversely, the Pb1692Δ*slyA* strain inhibition against Dd3937 exhibited no significant difference from that of wild-type Pb1692. The results imply a direct outcome of the overexpression of competition-related mechanisms following *phoP* deletion. Arguably, one of the major players in this increased *in planta* competition aggressiveness is the extensive array of 23 T6SS-related genes overexpressed in the absence of PhoP.

Both Pb1692Δ*slyA* and Pb1692Δ*phoP* strains next had their virulence against potato tubers examined by measuring the extent of maceration in early and late infection. The results showed that despite the positive regulation of T6SS- and PCWDE-encoding genes in the Pb1692Δ*phoP* mutant, this strain was attenuated in virulence, similar to the Pb1692Δ*slyA* strain, with no statistically significant difference between them (Fig. 4B). Compared to the bacterial competition assay, tissue maceration comprises a complex phenotype that encompasses a wide range of necessary factors. Thus, the transcriptional disruption of a large array of genes in the four regulons (PhoP-EI, SlyA-EI, PhoP-LI, and SlyA-LI), ranging between 68 and 317 protein-coding genes, may be responsible for the attenuated tissue maceration in these mutants (Table S2).

Aiming to inspect the possible PhoP-regulated mechanisms involved in this increased *in planta* growth inhibition by Pb1692 over that of Dd3937, we will next emphasize secretion system regulation. The T6SS is a protein complex assembled through both bacterial membranes, featuring a contractile sheath and an injectisome-like structure, composed of hemolysin-coregulated proteins (Hcp), that delivers specialized effectors into target cells (31). Both T6 structure- and effector-encoding genes were found under PhoP regulation, totaling 23 T6SS-related genes consistently overexpressed upon *phoP* deletion across the early and late infection (Table S2). By comparing evidence from the PhoP network in Pb1692 with that from previously reported analyses on a QS mutant strain of *P. atrosepticum* (*Pa*1043) during infection on potato tubers (21), some important observations were made. For this analysis, the fundamental difference between the two mutant strains, i.e., Pb1692Δ*phoP* and *Pa*1043Δ*expI* strains, is that the *expR1* regulator is overexpressed in the first and repressed in the second. In the *Pa*1043Δ*expI* strain, 23 T6SS genes exhibited a strong transcriptional decrease in *Pa*1043 upon the disruption of *expI*-*expR1* (Fig. 4C). On the contrary, Pb1692 overexpresses *expR1* along with 23 T6SS genes in the absence of *phoP*. This pattern shows the direct interference of QS disruption over the transcriptional modulation of T6SS genes in *Pa*1043. Additionally, it strongly suggests that the PhoP-dependent regulation of the T6SS gene cluster in Pb1692 primarily depends on the *expR1* regulator.

Moreover, we analyzed T3SS regulation in the two mutants (Fig. 4C). Curiously, both *phoP* and *expI* mutants exhibited a similar pattern of extensive downregulation of the T3SS genes. In Pb1692, the PhoP transcriptional impact encompasses 20 genes. Of these, 11 have detectable orthologs in *Pa*1043 under the transcriptional influence of QS. In this context, since *expR1* homologs are regulated in opposite directions in each mutant, the PhoP network in Pb1692 seemingly regulates several elements from the T3SS in a QS-independent manner.

### Characterizing new gene families within the T3SS operon in *Pectobacterium*.

During the secretion system regulation analyses, we found two GS genes unannotated by BLASTP-based methods within a T3SS-encoding cluster in Pb1692 (*AED-0001197* and *AED-0003294*). While *AED-0003294* was successfully detected under PhoP regulation (Table S2), *AED-0001197* exhibited slightly above-threshold false discovery rate (FDR) values and was not listed among the differentially expressed genes. Nonetheless, the *in planta* differential expression of *AED-0001197* in the *phoP* mutant strain could be confirmed through qRT-PCR (Fig. S2). By examining the sequences from their products, we found no detectable functional domains that indicated their biological role. Hence, aiming to classify these unannotated genes, we implemented sensitive sequence comparison techniques (see Materials and Methods for details) to examine their primary structure and search for conserved motifs. This analysis led to the identification of one novel motif in each protein, which are associated with (i) bacterial Ig-like (Big) and cadherin domains and (ii) *hrp*-related chaperones.

**FIG 5:**
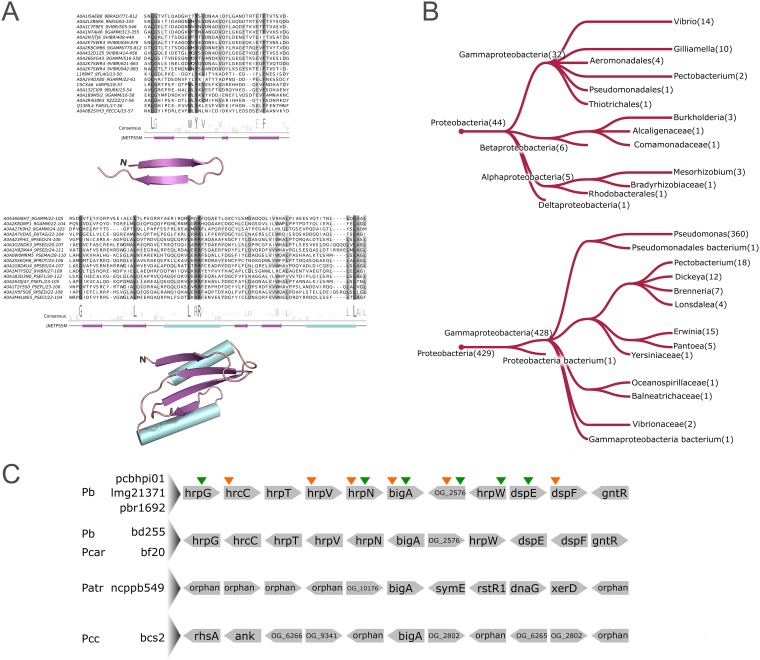
Multiple-sequence alignments, structural scaffolds, and phyletic distributions of two conserved motifs in type III secretion system-related putative effectors found in Pb1692. (A) The degree of conservation of each amino acid column is highlighted in increasingly darker shades of gray. Below each alignment, the amino acid logo depicts the amino acid consensus among the representative sequences. Secondary structure predictions next are displayed in both linear and folded (obtained through sequence modeling) configurations. Purple arrows represent β-sheets, and cyan cylinders represent α-helices. (B) Taxonomic groups in which the Big-associated (top) and HrpV-borne (bottom) motifs are found in sequences exhibiting the same domain architecture as that in Pb1692 are depicted in the phylogenetic trees. Taxonomic distributions were assessed through the Hmmsearch online tool (32). The branches are labeled with the corresponding number of genes found in the group. (C) Genomic architectures of seven *Pectobacterium* strains are represented, depicting five genes up- and downstream in *bigA* gene neighborhoods. Gene names were annotated according to sequence searches on the eggNOG database, where applicable. Sequences unannotated by eggNOG are either represented by their respective orthologous group labels (see Materials and Methods for details) or regarded as orphans in case no orthologous sequences were identified across the SRP genomes. Different species are abbreviated on the left: Pb (*P. brasiliense*), Pcar (*P. carotovorum*), Patr (*P. atrosepticum*), and Pcc (*P. carotovorum* subsp. *carotovorum*). Species names are adjacent to strain names. Arrows above the top architecture indicate up-/downregulation (up or down arrow, respectively) of the respective genes by PhoP in Pb1692 during either early infection (orange) or late infection (green) on potato tubers.

After extensive iterative searches against the UniprotKB online database via the Hmmer search tool (32) and sequence alignment inspections, we gathered 538 and 429 similar sequence sets for the Big-associated motif and the *hrp*-related chaperones, respectively. The Big-associated motif, found in AED-0003294, exhibits highly conserved Tyr and Leu residues in the second β-sheet and prior to the first β-sheet, which may represent core residues in their structure (Fig. 5A). This conserved motif was found primarily in sequences exhibiting no companion domains, similar to those from *Pectobacterium* genomes. Representative genes encoding this exact architecture are most common in *Gammaproteobacteria*, encompassing diverse lifestyles such as animal and plant pathogens (e.g., *Vibrio* and *Pectobacterium* genera) or insect gut symbionts (e.g., *Gilliamella* genus). Additionally, *Alpha*- and *Betaproteobacteria* also carry genes encoding the same architecture, including organisms from *Mesorhizobium* and *Burkholderia* genera (Fig. 5B). Aside from the instances in which these Big-associated motifs are found solo in a given sequence, they are even more frequently found in long (∼1,000 amino acids) proteins constituted by numerous domain repeats. These repeats often include combinations of Cadherin and/or Big domains (Pfam entries PF00028 and PF02369), along with the novel Big-associated motif. These families are known for their typical association with phage-like structures and have been linked to carbohydrate recognition on the cell surface, enabling phage adsorption (33), which will be further discussed below.

In the second unannotated gene product (AED-0001197), we found a conserved α_2_β_4_ structure, which was underpinned by 429 similar sequences gathered during the iterative searches described above (Fig. 5B). Approximately 45% of the significant hits found were annotated as HrpV, which form chaperone heterodimers with HrpG to regulate T3SS expression (34). Importantly, besides this newly found α_2_β_4_ motif, no other annotated domains could be detected in any of those 429 HrpV-like sequences. We next investigated the structural conservation of those sequences using HHpred (35), which detected similarities with type III chaperones, such as those from SchA or CesT (PDB entries 4G6T and 5Z38). Thus, although the primary structure does not match any currently annotated domain, the α_2_β_4_ fold found in these sequences was recognized as being similar to type III chaperone-related by HHpred. The impossibility of annotating the HrpV sequence via BLASTP-based methods may be a consequence of extensive sequence variations occurring in different organisms. This notion is supported by our orthology analysis, which has split the HrpV sequences from *Pectobacterium* (represented in 50/61 genomes) and *Dickeya* (represented in 39/39 genomes) into two separate groups (Table S4). Nonetheless, the hidden Markov model (hmm) profile consolidated in this analysis (see Materials and Methods for details) was successfully detected in both groups.

10.1128/mSystems.00650-19.7 TABLE S4 Genome-wide detection of two newly discovered motifs in HrpV (GLLR) and Big-associated (GWYN) in 100 SRP genomes through HMMER scan. Download Table S4, XLSX file, 0.02 MB. Copyright © 2020 Bellieny-Rabelo et al. 2020 Bellieny-Rabelo et al. This content is distributed under the terms of the Creative Commons Attribution 4.0 International license.

We next inspected gene neighborhoods of *hrpV* and the Big-associated (*bigA*) genes across SRP genomes. As expected, all the 89 *hrpV* genes from both ortholog groups, i.e., OG_3591 (*Pectobacterium*) and OG_3957 (*Dickeya*), are consistently surrounded by T3SS-related genes (Table S5). However, this pattern is not observed in *bigA* orthologs. Gene neighborhood screening of seven out of ten genomes carrying *bigA* orthologs (three genomes were not suitable for the analysis due to incompletion of genome assembly) revealed three different types of neighborhoods. Four Pectobacterium brasiliense strains and one strain of *P. carotovorum* exhibit *bigA* homologs within T3SS gene clusters (Fig. 5C). In one strain of *P. atrosepticum*, the *bigA* homolog is adjacent to phage elements, including homologs of the transcriptional repressor *rstR1*, the DNA primase *dnaG*, and the site-specific recombinase *xerD*. The strains of *P. carotovorum* subsp*. carotovorum* have their *bigA* homolog surrounded by unannotated genes and orphan genes that could not be clustered with any other sequences from SRP during the orthology analysis. Furthermore, the newly identified Big-associated gene *bigA*, described above, was predicted as a horizontally transferred gene (Fig. 3D). By inspecting the surrounding genes within the Pb1692 T3SS cluster, the *bigA* gene is the only one that exhibits full support from the HGT prediction (Fig. 3D and Table S3). This result suggests that *bigA* acquisition is posterior to the consolidation of the T3SS in the Pb1692 genome, which is consistent with the above-described genus-specific prediction for *bigA*. Together, these results elucidated the presence of HrpV and the HGT candidate *bigA* under PhoP regulation. Moreover, the *in planta* transcriptional regulation of a known T3SS regulatory element (i.e., *hrpV*) by PhoP points to an additional layer of control over T3SS, which will be addressed in the Discussion.

10.1128/mSystems.00650-19.8 TABLE S5 Gene neighborhood of *hrpV*-containing genes in 89 SRP genomes represented by domain architectures predicted by HMMER3. Download Table S5, XLSX file, 0.02 MB. Copyright © 2020 Bellieny-Rabelo et al. 2020 Bellieny-Rabelo et al. This content is distributed under the terms of the Creative Commons Attribution 4.0 International license.

## DISCUSSION

### The influence of lineage-specific rearrangements in the PhoP and SlyA regulatory networks on host adaptation.

It has been reiterated that the birth of novel functions in prokaryotes is mainly driven by HGT instead of by duplication and divergence (36, 37). For instance, it has been estimated that in representatives of both *Escherichia* and *Salmonella* genera, more than 98% of protein family expansions arise from HGT (37). Moreover, even gene copy increments in bacterial genomes can often be a consequence of HGT rather than duplication events (7, 38). As a consequence, the fixation of newly acquired genes requires that existing regulatory circuits reshape accordingly, allowing those new elements to fit properly into specific transcriptional programs. This often results in the recruitment of new genes into existing transcriptional networks (5). Our results showed PhoP, SlyA, and PecS exhibited a consistent bias toward the regulation of GS-HGT candidate genes at late plant infection. This indicates that transcriptional mobilization of recently acquired genes by these regulators plays a particularly important role in the late stages of infection in these organisms. These findings may even point to a broader trend in which these distinctive traits are preferentially recruited in late stages of the soft-rot disease. At this point in time, with the increasing availability of nutrients in consequence of plant cell lysis (14), the demand for mobilization of distinctive traits in closely related lineages could be specifically focused on interspecies competition.

The participation of PhoP in the regulation of GS genes was also reported in the *Salmonella* genus, as ∼50% of the genes found under PhoP regulation have no orthologous counterparts outside this group (39). This evidence is in accordance with the overrepresentation of GS genes found under PhoP control in Pb1692. It also reinforces the PhoP tendency toward regulating GS loci, despite the contrasting lifestyles between most *Salmonella* and *Pectobacterium* representatives. A similar pattern has been reported in Yersinia pestis and Y. enterocolitica in the RovA regulatory network (40). That report uncovered horizontally acquired genes within the RovA regulon, including genes associated with disease development (40). The *rovA* gene belongs to the MarR family of regulators, from which the best-characterized members, i.e., RovA and SlyA, have been linked to direct binding site competition with the histone-like nucleoid structuring regulator (H-NS) (41, 42). The H-NS role in the repression of horizontally acquired regions is widely characterized in Gram-negative bacteria, as it typically works through the recognition of AT-rich regions (43, 44). These observations converge with the results presented here, as 26.5 and 41.5% of all genes regulated by SlyA at early or late infection, respectively, were successfully predicted, by at least one parametric method, as HGT candidates.

Of particular importance, our results shed light on the relevance of transcriptional network rearrangement processes for PhoP network expansion, which includes the acquisition of other transcriptional regulators, such as *carR* and *expR1*. The evidence found here for the horizontal acquisition of *carR* and *expR1* is in accordance with previous phylogeny-based predictions of *luxI-luxR* horizontal transfer within the *Proteobacteria* phylum (45), as well as between Gram-negative and -positive bacteria (46). These two regulatory connections, i.e., *phoP*-*carR* and *phoP*-*expR*, represent an important innovation in the SRP group that impacts the regulation of several host adaptation-related systems in Pb1692 and possibly other species in the *Pectobacterium* genus.

### The *deoR1* transcription factor and the prominent role of SlyA in carbohydrate metabolism regulation.

**FIG 6:**
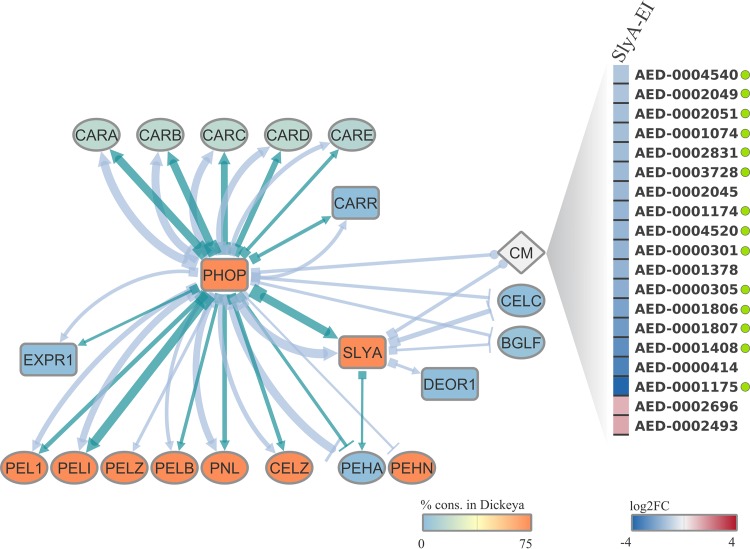
Regulatory interplay between PhoP, SlyA, and QS systems in Pb1692. Three subsets of host adaptation and/or virulence themes were extracted from both PhoP and SlyA *in planta* regulons. Transcriptional regulator genes in the network are highlighted in square shapes. The diamond shape represents an array of genes annotated by the KEGG term 09101, carbohydrate metabolism (CM). Each node is colored according to relative presence in the *Dickeya* genus according to the color scale at the bottom of the network. The target link shape depicts the link relationship between source and target nodes: upregulation (arrow), downregulation (perpendicular line), or mixed (circular shape). The source link shape is represented as a square to indicate “in the absence of,” which is the direct inference based on the RNA-Seq experiment performed using the two Pb1692Δ*phoP* and Pb1692Δ*slyA* mutants. Increasingly thicker links in the network represent higher log_2_ fold change (log2FC) values. Light-blue and dark-green links indicate regulation at early and late infection, respectively, by one of the two analyzed regulators (PhoP or SlyA). On the right side of the network, a one-column heatmap depicts the differential expression (log_2_ fold change) in genes associated with carbohydrate metabolism regulated by SlyA at early infection. Genes are represented by the respective locus tags according to the NCBI database. Green circles adjacent to individual genes highlight those for which PhoP-dependent regulation at early infection was also found.

As previously highlighted, the transcriptional regulator *deoR1* homologs spread across 53 out of 61 *Pectobacterium* genomes, whereas the *Dickeya* lineage apparently lost this gene. DeoR-type regulators have been recognized for a long time as transcriptional repressors of carbohydrate metabolism-related genes (47, 48). In the Gram-positive soilborne bacterium Corynebacterium glutamicum, the DeoR-type regulator SugR was shown to repress the transcription of phosphoenolpyruvate-dependent phosphotransferase system (PTS) genes, such as *ptsG*, *ptsS*, and *ptsF* (49). In this context, our data set unveiled an expression pattern following *slyA* deletion that corroborates this notion. While *deoR1* displays increased expression, four PTS permease-encoding genes (*AED-0001174*, *AED-0001290*, *AED-0002702*, and *AED-0003729*) consistently show decreased transcription in Pb1692 (see Table S2 and Fig S2 in the supplemental material). This could account for a Deor1-dependent repression of those permease-encoding genes. Another DeoR family member, termed UlaR, also was identified as a repressor of the l-ascorbate gene cluster (*ula*) in Escherichia coli; this repressor was shown to release the *ula* promoter region upon binding to the l-ascorbate-6-phosphate molecule (50). These reports are strikingly supported by our data set, in which, upon the overexpression of *deoR1* in the *slyA* mutant, 19 out of 21 genes annotated with the carbohydrate metabolism KEGG term (09101) within the SlyA regulatory network were consistently downregulated at early infection (Fig. 6). These observations suggest that SlyA directly or indirectly represses the *deoR1* homolog in wild-type Pb1692, which in turn may release DeoR1 from repressing an array of carbohydrate metabolism-associated genes. Importantly, 76.2% (16/21) of the carbohydrate metabolism-related genes repressed in SlyA-EI were also repressed in the PhoP-EI regulon (Fig. 6 and Table S2). Thus, since Pb1692Δ*phoP* and Pb1692Δ*slyA* mutant strains exhibit opposite patterns of *slyA* regulation, the evidence indicates that normal expression levels of both *phoP* and *slyA* are required for the expression of these carbohydrate metabolism-associated genes *in planta*.

### PhoP/QS interplay *in planta* in Pb1692: transcriptional regulation of carbapenem biosynthesis- and PCWDE-encoding genes.

Two out of three quorum-sensing regulator homologs from the Pb1692 genome were found to be under PhoP transcriptional regulation, *expR1* and *carR*. The absent *expR* homolog in the PhoP regulons is the *expR2*-*virR* gene, which has been shown to regulate several virulence themes, such as iron uptake, motility, and expression of PCWDEs in *P. atrosepticum* (51, 52). The mechanistic synergy between ExpR1 and ExpR2 has been previously described by Sjoblom et al. (29), in which both regulators were implicated in the control of virulence-related genes in *Pectobacterium*. In that study, they also observed the ability of ExpR2 to sense a broader range of autoinducer (AI) molecules than ExpR1 (29). Notably, QS has been previously reported to be controlled by other global regulators. For example, both in Pseudomonas syringae (53) and in Pb1692 (54), the relationship between the ferric uptake regulator (Fur) and the QS system was determined through contrasting concentrations of N-acylhomoserine-lactone produced by the *fur* mutant and wild-type strains. The regulation of QS by PhoQ/PhoP observed in P. fluorescens also seems to be affected by the Mg^2+^ concentration level in the medium (55).

The biosynthesis of the carbapenem antibiotic is an important QS-subordinate system. CarR-dependent QS regulation in Pectobacterium carotovorum subsp. *carotovorum* relies on the presence of N-(3-oxohexanoyl)-l-homoserine lactone (OHHL) ligand (28). Interestingly, the stability of both carbapenem and the OHHL molecules are affected by pH variations (56, 57). In this context, the PhoPQ two-component system ability to respond to pH variations (58, 59) may (at least partially) explain the success of recruiting *carR* and other *car* genes into the PhoP regulatory network. Thus, in the absence of *phoP*, *carR* and five other *car* genes are overexpressed *in planta*, which, by inference, means that the PhoP network suppresses their transcription *in planta* in the wild type. Hence, PhoP activation, arguably as a consequence of milieu acidification, is able to prevent carbapenem biosynthesis under pH conditions that are unfavorable for the antibiotic stability (Fig. 6).

A similar logic might also apply to the PhoP regulation of *expR1* and several PCWDEs. Indeed, we observed a marked contrast in PhoP regulation patterns over distinct PCWDE classes exhibiting (i) neutral/alkaline optimum pH (∼6.8 to 8), such as pectate/pectin lyases, and a cellulase (19, 20), and (ii) acidic optimum pH (∼6), such as polygalacturonases (Fig. 6) (60). This regulatory pattern does not correspond to the unidirectional regulation of PCWDE-encoding genes through the QS-RsmA system, which has been repeatedly reported in the *Pectobacterium* genus (16, 27). Thus, the fact that the PhoP network coerces the expression of different sets of PCWDE in opposite directions at the same stage implies that the QS-RsmA cascade is not the only regulatory mechanism controlling those genes in Pb1692. Therefore, although at first glance our results suggest a direct PhoP-ExpR1-RsmA-PCWDE regulatory hierarchy, an additional fine-tuning step enforced either directly or indirectly by PhoP seems to provide an alternative control system over PCWDE transcription. Moreover, this alternative pathway seems to be RsmA independent, which follows a previously proposed model of QS regulation (29), since neither *rsmB* nor *rsmA* transcription are influenced by PhoP absence in Pb1692. In this context, the regulation of PCWDE by PhoP in Dickeya dadantii has been previously reported. However, PhoP seemed to cause a unidirectional impact on pectate lyases and polygalacturonases (4), in contrast to what was observed in Pb1692. Such contrast should not be surprising, since the *Dickeya* spp. lack the *expR1* QS element, which may be an important part of the PhoP-dependent regulation of PCWDE in Pb1692. Thus, the different patterns found in these two genera may occur as a result of the PhoP-ExpR1 interplay in the regulation of PCWDE that seems to take place in *Pectobacterium* and not in *Dickeya* spp.

**FIG 7:**
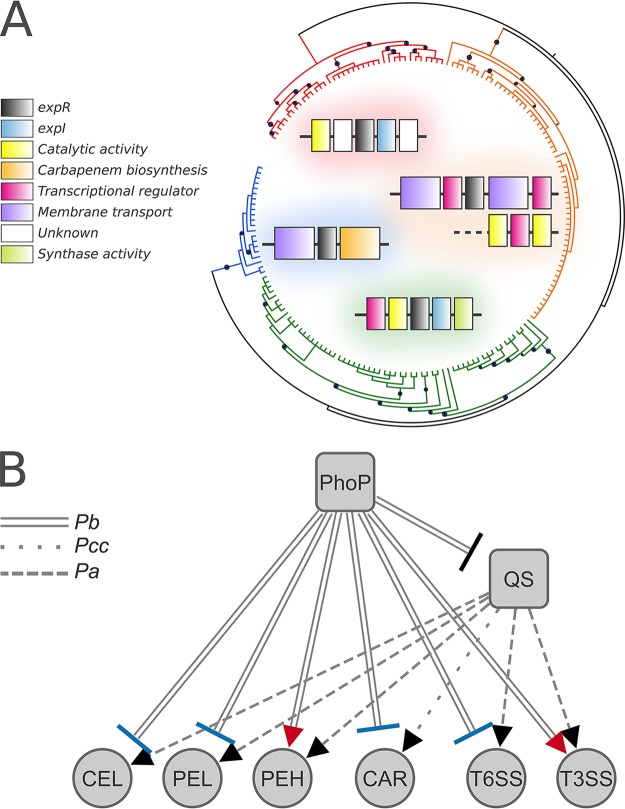
Evolution of ExpR subfamilies in distinct genomic contexts in SRP and the summary of PhoP-QS interplay in Pb1692. (A) The phylogenetic reconstruction of the autoinducer recognition module found in the ExpR/LuxR homologs from *Pectobacterium* and *Dickeya* was inferred by approximately maximum likelihood. The tree is represented in unscaled branches, with bootstrap values displayed as black circles. The different ExpR/LuxR orthologous groups previously predicted through OrthoMCL, all of which were clustered exclusively with sequences from the same genus, are highlighted in colors: ExpR2/VirR from *Dickeya* (red) and *Pectobacterium* (orange) genera, and ExpR1 (green) and CarR (blue) from *Pectobacterium*. For each group, the dominant gene neighborhoods found across the genus are depicted with the corresponding clade color in the background. (B) The summary network is displayed based on indirect inference from RNA-Seq: if a given gene/system is repressed in the absence of PhoP, then this gene/system is activated in the presence of PhoP. Blue and red target shapes in the network links represent correspondence and conflict between the observed regulation by PhoP in Pb1692 and the quorum-sensing regulation *in planta* reported in other *Pectobacterium* species, respectively. Regulated pathogenicity themes (abbreviations) are cellulases (CEL), pectate/pectin lyases (PEL), polygalacturonases (PEH), carbapenem biosynthesis (CAR), type VI and III secretion systems (T6SS and T3SS, respectively), and quorum-sensing regulators (QS). Link patterns represent the species on which the inference is based, Pectobacterium carotovorum subsp. *carotovorum* (Pcc), Pectobacterium atrosepticum (Pa), and Pb1692 (Pb).

Intriguingly, gene neighborhood analyses revealed that the *expR1* position in *Pectobacterium* genomes is locked upstream of *expI*, which is the exact same pattern as that observed in the *expR2*-*virR* homologs across the *Dickeya* genus (Fig. 7A). In terms of conservation, *expR2*-*virR* can be found in 100% of the SRP strains analyzed, and in *Pectobacterium* genomes, it is mostly surrounded by membrane transport systems and other transcriptional regulators, whereas the third and evolutionary more distant homolog, *carR*, exists in only 21 out of 61 *Pectobacterium* strains analyzed and is consistently adjacent to the carbapenem biosynthetic cluster in the genus, as previously reported by Shyntum et al. (61) (Fig. 7A). This agrees with a recent estimate that ∼76% of ExpR/LuxR genes exist in genomes without an adjacent ExpI/LuxI homolog (25). Also, according to the HGT predictions, the acquisition of *expR1* and *carR* by Pb1692 appears to have occurred through horizontal transfer in the *Pectobacterium* lineage. Although some species, such as *P. parmentieri* and *P. wasabiae*, lost the entire carbapenem biosynthesis cluster, as previously communicated by Shyntum et al. (61), *expR1* remains prevalent in the *Pectobacterium* genus. Thus, the evidence suggests that each of the three *expR*-*luxR* homologs found in *Dickeya* and *Pectobacterium* genera have been acquired horizontally in an independent fashion. Furthermore, horizontal acquisition of *expR*-*luxR* homologs has been predicted to occur either as a regulatory cassette that includes *expI*-*luxI* or individually (45, 62). This notion converges with the results observed in (i) *expR2*-*virR* or *carR* found in *Pectobacterium* genomes, in which individual acquisition is the most parsimonious assumption, and (ii) *expR2*-*virR* in *Dickeya* or *expR1* in *Pectobacterium* genomes, which most likely were acquired as a regulatory cassette (Fig. 7A).

Based on previous reports on how QS regulators modulate either PCWDE (21, 63) or carbapenem biosynthesis (28, 64) in SRPs, the possible PhoP-QS interplay can be inferred from our results. Hence, for pectate lyases (Pel), cellulase (Cel), and the *car* genes, the observed regulation of QS by PhoP agrees with those from previous reports, which reinforces the idea of QS regulatory mediation for those genes (Fig. 7B). Conversely, since two polygalacturonase-encoding genes exhibit the opposite transcriptional behavior, they may be controlled by a QS-independent mechanism under the PhoP network. This raises the possibility of a cascade within the PhoP network, providing additional pH-dependent control over the transcription of some PCWDE, including *Pectobacterium*-exclusive PCWDE, which seems to override QS regulation.

### The PhoP interplay with QS in the transcriptional regulation of T3SS and T6SS.

The role of T3SS in pathogenicity has been widely explored in a range of taxa, including some from the SRP group. In Pectobacterium atrosepticum, a significant reduction in virulence toward potatoes was observed in mutant strains lacking both T3SS structural genes *hrcC* and *hrcV* (65). In that same study, Holeva et al. (65) also observed that upon the deletion of either the T3SS-related effector *dspE* or the helper gene *hrpN*, virulence was also reduced. Among the 12 T3SS-related genes found under PhoP network control in Pb1692 at early infection, *hrcC*, *hrcV*, and *hrpN* are present (Table S2). This correspondence implies that the results observed in our virulence assays, in which the Pb1692Δ*phoP* mutant exhibited attenuated virulence compared to that of the wild type, were (at least partially) impacted by the repression of those T3SS-related genes.

The transcriptional regulation of T3SS involves complex coordination by several regulatory systems, and some have been reported in different bacterial lineages. This includes the QS system (21) as well as other regulators, such as SlyA (66), PhoP (67), PecS (9), and Fur (68). Specifically in Salmonella enterica, the T3SS termed Spi/Ssa was observably under indirect PhoP control through the SsrB/SpiR system (67, 69). Otherwise, within the SRP group, previous efforts have concluded that PhoP deletion in Dd3937 has no impact on the regulation of T3SS under *in vitro* conditions (70). In contrast with this observation, our results show a wide impact exerted *in planta* by PhoP on T3SS transcription. This contrast indicates that PhoP regulation over T3SS transcription depends on specific cues that cannot be met *in vitro*. However, since a PhoP-T3SS regulatory link has not been assessed *in planta* in Dd3937 so far, which would allow a direct comparison with the results observed in Pb1692, there is not enough data currently available to predict if (i) the PhoP-dependent regulation of T3SS *in planta* is orthologous among Pb1692 and Dd3937 or (ii) this mechanism evolved at some point within the *Pectobacterium* lineage. Nonetheless, PhoP control over T3SS transcription *in planta* observed in Pb1692 had not been reported in phytopathogens so far. Moreover, our SlyA regulon analysis also shows that contrary to what was reported in Dd3937 by Zou et al. (66), Pb1692 does not rely on SlyA to control T3SS transcription.

Unlike the T3SS, the role of the T6SS in plant colonization processes is not deeply understood in phytopathogens and specifically within the SRP group. However, it has been reported that the T6SS, similar to the T3SS, is under the regulatory control of the QS, as observed in Pseudomonas aeruginosa (71) and in the *P. atrosepticum* mutant strain lacking the *expI* gene (21). The T6SS has been functionally implicated in both host defense manipulation and interbacterial competition. However, these two functionalities are not characteristic of any of the five T6SS phylogenetic clades recently reported and, thus, cannot be used to separate T6SS phylogenetic groups (72). As an example, the ability of a T6SS-related Hcp protein to facilitate tumorigenesis in potatoes has been reported in the phytopathogenic Agrobacterium tumefaciens DC58 (73). In the same species, the homologous T6SS was later implicated in intra- and interspecies competition *in planta* (74). Furthermore, T6SS has been specifically reported as a decisive asset in interspecies competition *in planta* for Pb1692 (61). In agreement with previous reports, our results from *in planta* bacterial competition indicate that the increased transcription of T6SS as a consequence of *phoP* deletion in Pb1692 is an important factor to boost competitive advantage against closely related competitors (Fig. 4A and C).

The observed influence of QS over T6SS transcription in *P. atrosepticum* spans 23 genes, which exhibited decreased transcription intensity upon QS disruption (21). Similarly, T3SS transcription was also impacted by the disruption of QS, as 21 genes were consistently repressed (21). We verified that in the absence of PhoP, the QS receptor *expR1* is overexpressed, and so are several T6SS-related genes, which strongly suggests that QS mediates PhoP regulation over T6SS during infection. Indeed, this hypothesis is supported by results from Liu et al. (21) in *P. atrosepticum*, in which T6SS transcription decreases as the QS system is disrupted (Fig. 7B). On the other hand, since T3SS exhibits the opposite regulatory pattern, similar to the one observed in *P. atrosepticum* following QS disruption, it is likely that PhoP does not depend on QS to regulate the T3SS. Hence, PhoP must be able to override QS regulation of T3SS-encoding genes and control their expression. This could result either from PhoP directly binding to their promoter regions or through an alternative QS-independent regulatory cascade.

### New T3SS-related families and the transcriptional regulation of the *hrp* gene cluster by PhoP.

The bacterial Ig-like domains are highly promiscuous entities that have been described in structures of (i) adhesins, such as invasins and intimins (75, 76), (ii) phage-tail proteins (77), and (iii) bacterial surface glycohydrolases (78). The presence of Big-encoding genes in bacteria has been associated with horizontal transfers as a result of its frequent presence in phage genomes (78), which was strongly corroborated by our HGT prediction. Although the precise function of Big domains remains elusive, several lines of evidence point to a role in surface carbohydrates recognition (77, 78). Indeed, from the guilt by association standpoint, this could be the case for these newly found BigA proteins, since they are located in a region where T3SS-related products are typically encoded. Also, the analyses conducted in this study support the posterior horizontal acquisition of the *bigA* gene in Pb1692 independently from the rest of the T3SS gene cluster.

The other newly found motif in T3SS-related proteins from Pb1692 is comprised of an α_2_β_4_ structure, which was found to be similar to that of bacterial HrpV. Curiously, this motif has been identified before in Erwinia amylovora, although it has not been deposited in any public domain databases (34). HrpV is known as a negative regulator of the *hrp*-*hrc* gene cluster in P. syringae (79). One of the important protein interactions involving HrpV is the ability to bind HrpS. This interaction forms the HrpV-HrpS complex, which blocks the formation of HrpR-HrpS heterohexamers, subsequently hindering HrpS’s ability to bind and activate the *hrp*-*hrc* promoter (80, 81). This negative regulation imposed by HrpV was shown to be attenuated by its interaction with HrpG, which then generates a double-negative regulatory circuit controlling the expression of T3SS elements (82). We also found that, apart from *hrpV*, none of the other transcriptional regulators of the T3SS region (i.e., *hrpG*, *hrpS*, *hrpR*, and *hrpL*) are affected by *phoP* deletion, yet 20 *hrp*-*hrc* genes exhibit differential expression in the PhoP mutant strain. Thus, the expected result of having the main repressor of *hrp*-*hrc* (i.e., *hrpV*) transcription repressed should be the increased expression of several genes in this region. However, the opposite was observed, as 20 T3SS genes are consistently repressed across early and late infection stages. These observations imply that an alternative regulatory mechanism employed by the PhoP network coordinates the expression of T3SS elements, one that is independent of HrpG/S/R/L. As previously discussed, QS does not seem to be the mechanism responsible for this regulation, based on the results from PhoP regulon analyses combined with past observations (21). This means that PhoP is either directly or indirectly involved in the regulation of T3SS, apparently independently from Hrp-borne regulators or the QS system. Indeed, this hypothesis adds complexity to the topic if taken together with the conclusions reported by Bijlsma and Groisman (67) in Salmonella enterica, in which PhoP is responsible for the posttranscriptional regulation of a T3SS gene cluster.

By elucidating the mechanisms of network expansion through transcriptional rearrangement in an important phytopathosystem, we found a wide range of host adaptation- and environmental fitness-related traits being coopted by SlyA and especially by PhoP. Here, we uncover the success of the centralizing strategy that recruited carbohydrate metabolism regulation along with virulence-related systems controlled by QS in Pb1692 into the stress response regulator PhoP. This seems to provide optimized control over specific systems that may be sensitive to certain environmental conditions that can be recognized by the PhoQ/PhoP two-component system.

## MATERIALS AND METHODS

### Growth conditions and construction of *Pectobacterium brasiliense* 1692 mutant strains.

The strains and plasmids used are listed in Table S6 in the supplemental material. Luria-Bertani (LB) broth and agar plates were used for growing all bacterial strains at 37°C. Different antibiotics were used to supplement the media, i.e., kanamycin (50 μg/ml) and ampicillin (100 μg/ml) (Sigma-Aldrich), when needed. All reagents were used according to the manufacturer’s instructions. To generate the mutant strains, the ASAP database BLASTN tool was used to identify the Pb1692 *phoP* and *slyA* genes (*AED-0004376* and *AED-0001000*). Both Pb1692Δ*phoP* and Pb1692Δ*slyA* mutants were generated using a strategy developed by Datsenko and Wanner (83). Briefly, the up- and downstream regions flanking the target genes (approximately 1,000 bp) were amplified using specific primers (Table S7) using PCR. The kanamycin resistance gene cassette was amplified from the pKD4 plasmid. The resulting upstream fragment, kanamycin cassette, and downstream fragments were fused using primers denoted as previously described by Shyntum et al. (61). The fused PCR product was purified and electroporated into electrocompetent Pb1692 cells with pkD20. The resulting transformants were selected on nutrient agar supplemented with 50 μg/ml kanamycin. The HiFi HotStart PCR kit (KAPA system) was used in all PCRs. The PCR conditions were set as initial denaturation at 95°C for 3 min, followed by 25 cycles of denaturing at 98°C for 30 s, annealing at 60 to 65°C (depending on the primer set), extension at 72°C for 2 min, and a final extension at 72°C for 2 min. The Pb1692Δ*phoP* and Pb1692Δ*slyA* mutant strains were verified by PCR analyses and nucleotide sequencing.

10.1128/mSystems.00650-19.9 TABLE S6 List of bacterial strains and plasmids used in this study. Download Table S6, XLSX file, 0.01 MB. Copyright © 2020 Bellieny-Rabelo et al. 2020 Bellieny-Rabelo et al. This content is distributed under the terms of the Creative Commons Attribution 4.0 International license.

10.1128/mSystems.00650-19.10 TABLE S7 PCR primers used in this study. Download Table S7, XLSX file, 0.01 MB. Copyright © 2020 Bellieny-Rabelo et al. 2020 Bellieny-Rabelo et al. This content is distributed under the terms of the Creative Commons Attribution 4.0 International license.

### Complementation of *phoP* and *slyA* mutants.

The p-JET1.2/blunt cloning vector was used for the complementation of mutant strains. The *phoP* and *slyA* genes from Pb1692, including ∼500 nucleotides of downstream sequence containing the putative promoter sequence, were amplified by PCR using complementation primers listed in Table S7. The corresponding fragments were excised from an agarose gel and purified using the Thermo Scientific gel extraction kit according to the manufacturer’s instructions. The fragments were cloned into p-JET-T to generate pJET-*phoP* and pJET-*slyA* (Table S6). These plasmids were electroporated into electrocompetent Pb1692Δ*phoP* and Pb1692Δ*slyA* mutant strains, transformants (Pb1692Δ*phoP*-p*phoP* and Pb1692Δ*slyA*-p*slyA*) were selected on agar plates supplemented with 100 μg/ml ampicillin, and the cloned *phoP* and *slyA* were confirmed using PCR and sequencing.

### Tissue maceration assay and total RNA extraction from potato tubers.

Solanum tuberosum (cv. Mondial, a susceptible cultivar) potato tubers were sterilized with 10% sodium hypochlorite, rinsed twice with double-distilled water, air dried, and then stabbed with a sterile pipette to a 1-cm depth. A 10-μl aliquot of the bacterial cells at an optical density at 600 nm (OD_600_) equivalent to 1 were pipetted into generated holes. As a control, 10 mM MgSO_4_ was inoculated into potato tubers. Holes were sealed with petroleum jelly. Potato tubers were then placed in moist plastic containers and incubated for 12 and 24 h at 25°C. The macerated tissue was scooped and weighed 12 and 24 h postinoculation to quantify the extent of tuber maceration caused by Pb1692 wild-type and Pb1692Δ*phoP* and Pb1692Δ*slyA* mutant strains, as well as Pb1692Δ*phoP*-p*phoP* and Pb1692Δ*slyA*-p*slyA* complemented mutant strains. This experiment was performed in triplicates three independent times.

For RNA extraction, potato tubers inoculated with Pb1692 wild-type, Pb1692Δ*phoP*, and Pb1692Δ*slyA* strains were incubated at 25°C with high humidity in plastic containers for 12 and 24 h maximum. Sampling was done by scooping out macerated tissue. Macerated potato tissue from each inoculated site was scooped out and homogenized in double-distilled water. Bacterial cells were recovered by grinding the scooped macerated potato tissues in 20 ml of double-distilled water using an autoclaved pestle and mortar. Starch material was removed by centrifuging the ground tissues at 10,000 rpm for 1 min. The supernatant was removed and transferred into new sterilized 50-ml Falcon tubes containing RNA stabilization buffer (Qiagen, Hilden, Germany). The experiments were performed using three biological replicates, with three tubers per replicate.

### Determination of total RNA quality.

The concentration and purity of each extracted total RNA sample were evaluated using spectrophotometric analysis (NanoDrop ND-1000; NanoDrop Technologies, Wilmington, DE) at a ratio of 230 to 260 nm. The integrity of the RNA was further confirmed using 1% (wt/vol) agarose gel electrophoresis using 1% Tris-acetate-EDTA buffer at 100 V for 30 min and visualized, and an image was developed using a Gel Doc EZ system (Bio-Rad Laboratories, Berkeley, CA, USA). Using an Agilent 2100 Bioanalyzer (Agilent Technologies, Inc.), total RNA sample concentration, RNA integrity number (RIN), and 28S/18S ratio were determined.

### RNA sequencing, read mapping, differential expression analysis, and genome-wide functional annotation.

RNA samples were sequenced in the Novogene facility (Chula Vista, CA) using an Illumina NovaSeq 6000 machine. Reads sequencing quality analysis was carried out by utilizing FastQC software (https://www.bioinformatics.babraham.ac.uk/projects/fastqc). Low-quality segments were then trimmed by Trimmomatic v 0.36 (84). Trimmed reads were next supplied to *hisat2* v 2.1.0 (85), which performed read alignment to the reference genome of Pb1692 (WPP1692 version 3), obtained from the ASAP database (https://asap.genetics.wisc.edu/). The number of aligned reads was subsequently computed by the *featureCounts* package (86), and differential expression was analyzed by utilizing the EdgeR package (87), both within the R environment (https://www.r-project.org/). Genes exhibiting transcriptional variation of log_2_ fold change of >1 (upregulation) or <−1 (downregulation), with FDR of <0.05, were designated differentially expressed. Comparisons of mutant samples to wild-type ones were done at 12 and 24 hpi. Pb1692 sequences were functionally annotated by using the eggNOG-mapper tool (88). The annotation provided by eggNOG was next used to retrieve higher annotation levels in the KEGG library hierarchy (KEGG B and A) (89) through custom scripts written in the Perl language (https://www.perl.org/). Enrichment of KEGG terms was determined by one-tailed Fisher exact test, and subsequent *P* value adjustment by FDR (*q* value of <0.05) was performed by custom R scripts using the Pb1692 genome as the background data set. An additional level of annotation was provided by conserved domain inspection on the protein sequences carried out using HMMER3 (90) and the Pfam database (91). Venn diagrams were created using the InteractiVenn online tool (92).

### Genus-specific contents, enrichment in specific regulons, and genome simulations.

The framework used to identify orthologs among strains from *Pectobacterium* and *Dickeya* genomes was previously described (6). Briefly, genomic and proteomic information was acquired from the NCBI online database for 61 and 39 *Pectobacterium* and *Dickeya* strains, respectively. All protein sequences were clustered by implementing the OrthoMCL pipeline (93). The presence of representatives from each genus was analyzed throughout the 10,635 orthologous groups using custom Perl scripts. Those gene products that (i) belong to clusters populated exclusively with sequences from one of the genera or (ii) are orphans (not clustered) then are considered genus specific. To evaluate the possibility of overrepresentation of GS genes within individual regulons, we used both statistical analysis and computational simulations. Statistical verification performed in the R environment (https://www.r-project.org/) was based on a two-tailed Fisher exact test to assess the correlation between GS occurrence in a given regulon, using the respective strain genome as the background. Further, the computational simulations were performed to generate 10,000 shuffled copies of the respective genomes (Pb1692 or Dd3937 pseudogenomes) for each regulon comparison. In these comparisons for a single regulon, gene positions relative to each regulated gene were retrieved. These exact positions next were checked in each of the 10,000 pseudogenomes for the occurrence of GS genes. The total number of GS genes found in this assessment for each pseudogenome is computed and compared to the real data.

### Gene expression analysis through qRT-PCR.

To validate the differential expression analysis of genes from the RNA-Seq data, a qRT-PCR analysis was performed using 12 selected genes. RNA samples used in the qRT-PCR analysis were the same as those used in RNA sequencing and were prepared using an RNeasy minikit (Qiagen, Hilden, Germany) according to the manufacturer’s instructions. The first-strand cDNA was reverse transcribed from 5 μg of total RNA with the SuperScript IV first-strand synthesis system (Invitrogen). qRT-PCR was performed using a QuantStudio 12K Flex real-time PCR system (Applied Biosystems). Primers used in this study are listed in Table S7. The following cycling parameters were used: 95°C for 3 min, followed by 40 cycles of 95°C for 60 s, 55°C for 45 s, and 72°C for 60 s, and then 72°C for 7 min. *ffh* and *gyrA* (housekeeping gene control) were used to normalize gene expression. The gene expression levels of the following genes were analyzed: *pehA* (*AED-0002061*), *pehN* (*AED-0000675*), *expR1* (*AED-0000069*), *carR* (*AED-0003542*), *deoR1* (*AED-0001096*), *tssE* (*AED-0001996*), *tssC* (*AED-0001994*) *bigA* (AED-0003294), *hrpV* (AED-0001197), *celC* (*AED-0001290*), *bglF* (*AED-0003729*), and *AED-0001174*. The same method was applied to measure the expression levels of the *phoP* gene in the Pb1692 wild type. Overnight cultures of the wild type were inoculated into sterile potato tubers and extracted every 4 h for a period of 24 h (see “Tissue maceration assay and total RNA extraction from potato tubers,” above). For statistical analysis of relative gene expression, the threshold cycle (*C_T_*) method was used (94). A *t* test was used to determine statistical significance (*P* < 0.05).

### *In planta* competition assays in potato tubers.

Competition assays were performed in potato tubers as described by Axelrood et al. (95). In summary, Solanum tuberosum (cv. Mondial, a susceptible cultivar) tubers were sterilized with 10% sodium hypochlorite, rinsed twice with double-distilled water, air dried, and then stabbed with a sterile pipette to 1-cm depth. Overnight bacterial cultures of Pb1692 wild-type, Pb1692Δ*phoP*, Pb1692Δ*phoP*-p*phoP*, Pb1692Δ*slyA*, and Pb1692Δ*slyA*-p*slyA* strains and Dickeya dadantii at an OD_300_ equivalent to 0.3 were mixed in a 1:1 ratio and inoculated into surface-sterilized potato tubers. Holes were sealed with petroleum jelly. Potato tubers were then placed in moist plastic containers and incubated for 24 h at 25°C. Macerated tuber tissue was scooped out, and the CFU per milliliter of surviving targeted bacteria was determined by serial dilutions on LB supplemented with gentamicin (15 μg/ml). The number of colonies observed was then converted to CFU per milliliter. This experiment was performed in triplicates three independent times.

### Sequence analysis and protein domain characterization.

Orthologous sequences of unannotated proteins (Big-associated and HrpV) from SRP strains were aligned using Clustal Omega (96). The resulting alignments then were used in iterative searches against the UniprotKB database (www.uniprot.org) through Hmmersearch (32). The aligned results from the iterative searches were manually curated through Jalview alignment viewer (97), and the conserved areas in the alignments were selected. Those selected conserved blocks next were analyzed for secondary structure prediction and programmatic gap removals by Jpred (98). The resulting concise alignments were submitted to HHpred (35) to identify appropriate templates for structure modeling. This strategy managed to retrieve several PDB structures to be used as templates. These templates were selected for subsequent modeling according to the predicted probability (>40%).The following PDB entries were selected: for the Big-associated sequences, 5K8G, 4N58, 4G75, 4FZL, 5K8G, 4FZM, and 2XMX; for HrpV, 4G6T, 3KXY, 5Z38, 3EPU, 4GF3, 1JYA, 1S28, 5WEZ, 4JMF, and 1XKP. The models were predicted by Modeller (99), and visualization of these models was generated through PyMOL (100).

### Horizontal gene transfer prediction and regulatory network analyses.

Parametric methods are aimed at evaluating sequence composition bias in genes or genomic regions and measure their distance to the overall trend observed in the respective genome. These methods are especially powerful when applied in recent HGT candidate predictions, contrary to phylogenetic methods (101). Predictions were made with the support of two different methods, namely, GC content at the third codon position (GC3) and dinucleotide frequencies (DINT). The method choice was adapted based on the conclusions drawn in the benchmark conducted by Becq et al. (30). Genome-wide prediction of GC3 indexes of Pb1692 and Dd3937 coding sequences was made through the codonW tool (http://codonw.sourceforge.net/). Dinucleotide frequency analyses were performed using the *fasta2kmercontent* script from the CGAT package (102). The Kullback-Leibler distance (KLD) (103) was used to measure the difference between dinucleotide frequencies of individual coding genes and the respective genomes (averaged value for all coding sequences). KLD is calculated according to the following formula:

$$\mathrm{KLD}\left[p\left(g\right)∥p(G)\right]=\overset{N}{\underset{i}{\sum }}p(g_{i})\text{ log}\frac{p(g_{i})}{p(G_{i})}$$

*p*(*g*) is the probability distribution of dinucleotide frequencies in an individual coding gene, and *p*(*G*) is the probability distribution of averaged dinucleotide frequencies from all coding genes in that same genome. KLD values were computed in the R statistical environment. These two metrics (GC3 and DINT_KL) are then combined with the information on the genus specificity of genes previously collected from orthologous clustering to predict HGT candidates. The above-threshold genes that were also genus specific according to the previous analysis are predicted to be HGT candidates. Gene networks were analyzed using Cytoscape (104) and the heatmap generated with Gitools (105).

### Data availability.

The data sets generated in this study are available at the Sequence Read Archive (SRA; https://www.ncbi.nlm.nih.gov/sra) under the accession number PRJNA565562.
