# Supplementary figures and images for: Horizontally Acquired Quorum-Sensing Regulators Recruited by the PhoP Regulatory Network Expand the Host Adaptation Repertoire in the Phytopathogen Pectobacterium brasiliense
Source: mSystems. 2020 Jan 28;5(1):e00650-19. doi: 10.1128/mSystems.00650-19 (PMC6989131; doi:10.1128/mSystems.00650-19)

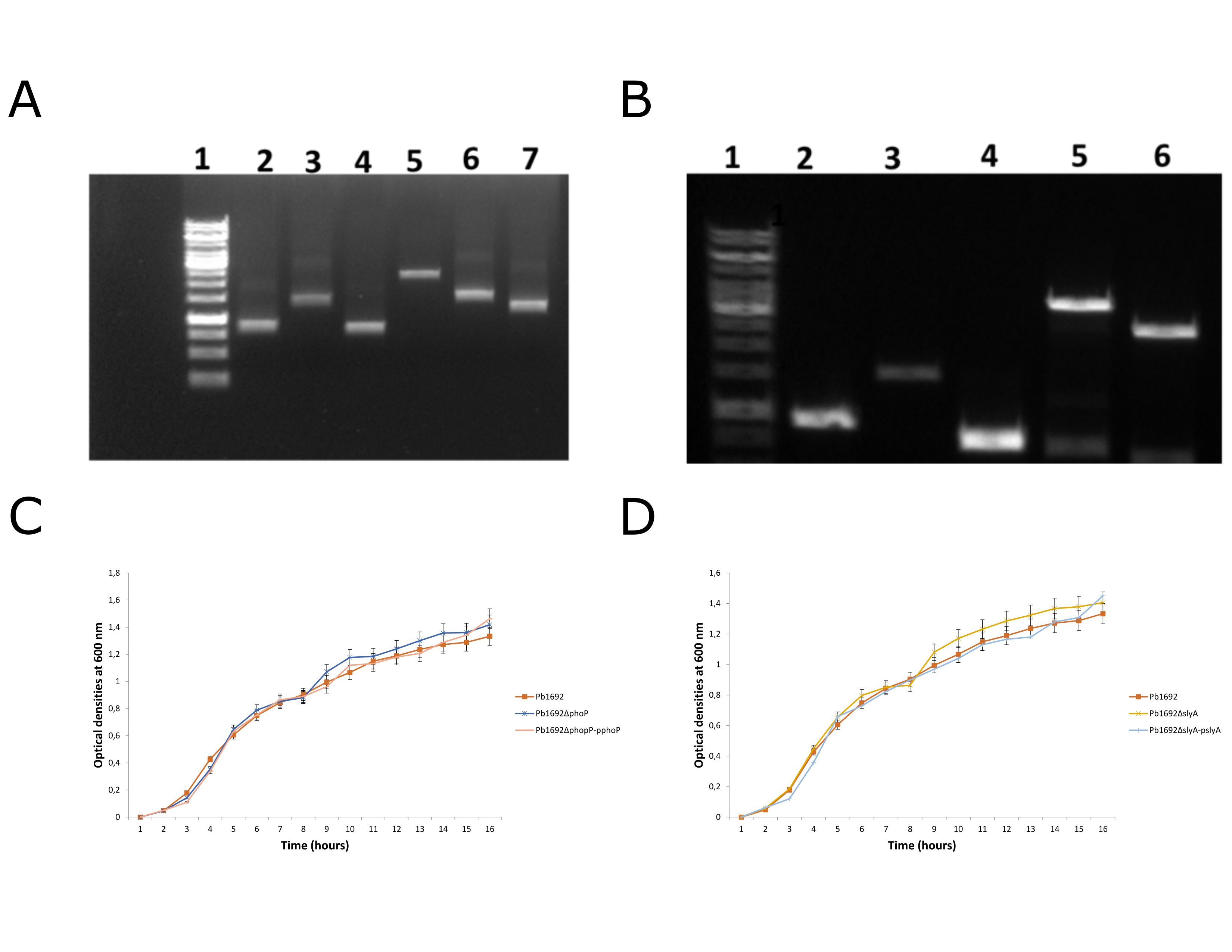

Supplement: FIG S1 [file mSystems.00650-19-sf001.tif]

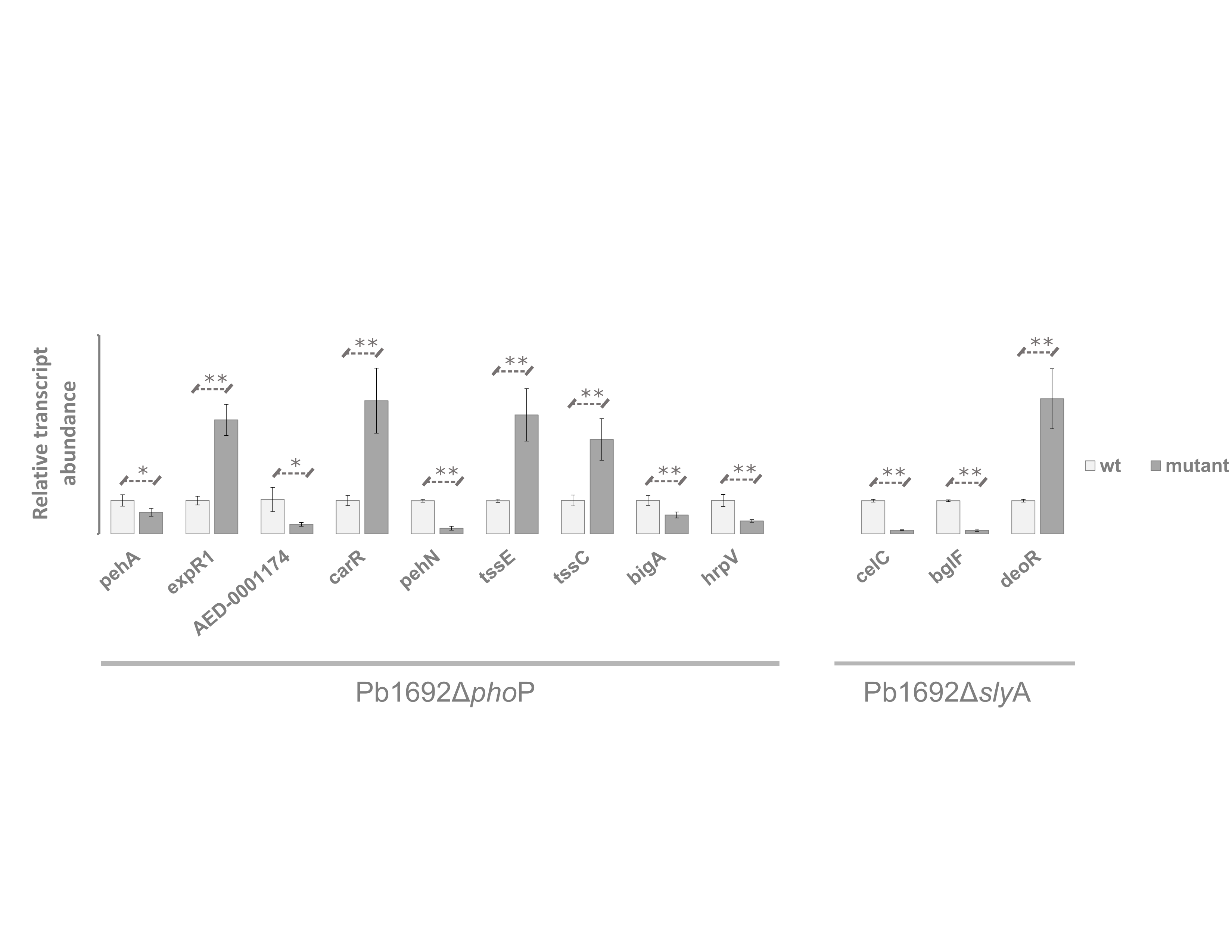

Supplement: FIG S2 [file mSystems.00650-19-sf002.tif]

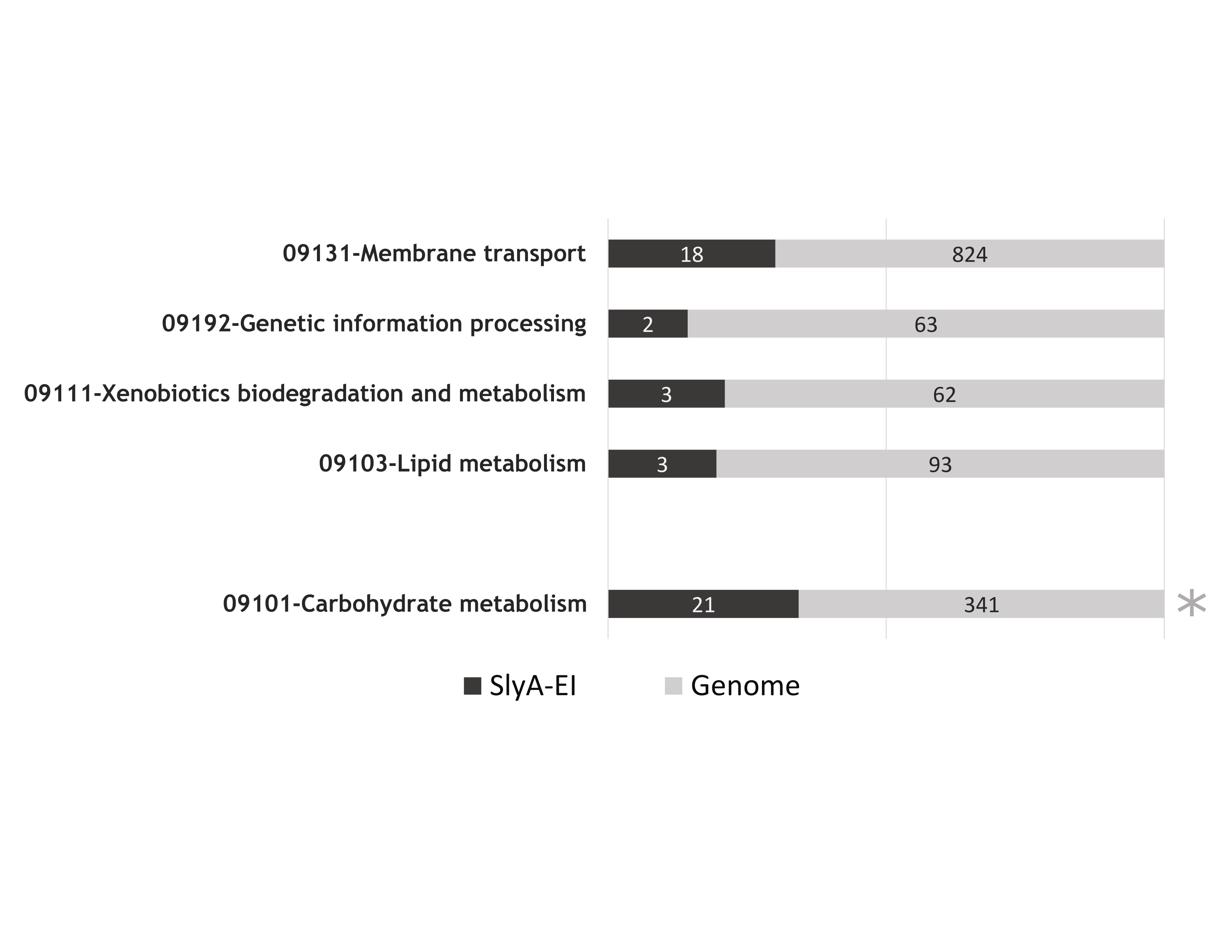

Supplement: FIG S3 [file mSystems.00650-19-sf003.tif]
